# Three members of the yeast N-BAR proteins family form heterogeneous lattices *in vivo* and interact differentially with two RabGAP proteins

**DOI:** 10.1038/s41598-020-58606-2

**Published:** 2020-02-03

**Authors:** Magali Prigent, Julien Chaillot, Hélène Tisserand, Emmanuelle Boy-Marcotte, Marie-Hélène Cuif

**Affiliations:** 1 Université Paris-Saclay, CEA, CNRS, Institute for Integrative Biology of the Cell (I2BC), 91198 Gif-sur-Yvette, France; 2 Institut sur la Nutrition et les Aliments Fonctionnels (INAF), Université Laval, 2440 Boulevard Hochelaga, Québec, QC G1V 0A6 Canada

**Keywords:** Endocytosis, Membrane curvature

## Abstract

The yeast N-BAR (Bin/Amphiphysin/Rvs167) protein Rvs167 is recruited by the Rab GTPase Activating Proteins (RabGAP) Gyp5 and Gyl1 to the tip of small buds to act in exocytosis. Investigating other N-BAR proteins involved in Gyp5/Gyl1/Rvs167 complexes, we found that Rvs161, an Rvs167 paralog, is absent from the complexes formed at the tip of small buds. Immunoprecipitation and Bimolecular Fluorescence Complementation (BiFC) analysis show that both Rvs167 and Rvs161 interact *in vivo* with Gvp36, an N-BAR protein. Rvs167 molecules also interact independently of Rvs161 and Gvp36. Rvs167/Rvs167 and Rvs167/Gyp5 interactions predominate over other combinations at the tip of small buds, suggesting that N-BAR lattices enriched in Rvs167 molecules form at these sites. By combining BiFC with markers specific to each organelle, we analyzed systematically in living cells the locations of the BiFC signals generated by combinations of the three N-BAR proteins. We show that the BiFC signals differ according to organelle and cell site, strongly suggesting heterogeneity in the composition of N-BAR protein lattices *in vivo*. Our results reveal that the organization of N-BAR protein lattices *in vivo* is complex and are consistent with N-BAR proteins forming various types of dimers and lattices of variable composition.

## Introduction

The BAR (Bin/Amphiphysin/Rvs167) domain superfamily is made up of highly conserved domains formed of dimeric alpha-helix coiled-coils. BAR domains can interact with membranes electrostatically: the positive charges of their surface interact with the negative charges of lipid bilayer headgroups. BAR proteins act as scaffolds to force membrane curvature and are involved in a variety of cellular processes requiring membrane shaping^1^. Several types of BAR proteins induce phosphoinositide clustering, promoting the formation of PI-enriched microdomains on their target membranes^2^.

The BAR protein superfamily includes N-BAR proteins, characterized by an additional N-terminal amphipathic helix which inserts into one leaflet of the membrane and promotes membrane curvature and dimerization^3,4^. Cryo-EM experiments with full-length endophilin and amphiphysin, two mammalian N-BAR proteins^5,6^, found that arrangement of dimers of the protein form polymer lattices on membranes *in vitro*. The formation of N-BAR lattices *in vivo* has not formally been demonstrated, but their existence is generally accepted because it fully explains the observed role of N-BAR proteins in membrane modeling.

**Figure 1:**
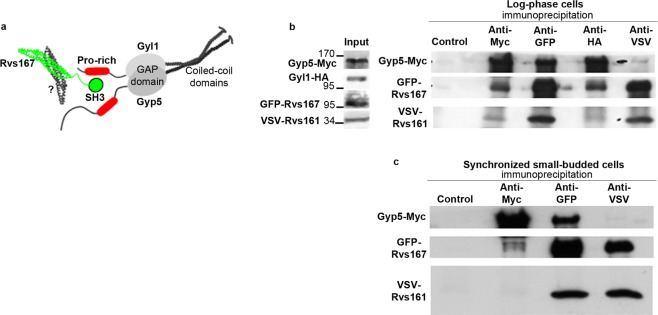
Rvs167, but not Rvs161, co-immunoprecipitates with Gyp5 in small-budded cells. (**a**) The RabGAP proteins Gyp5 and Gyl1 form heterodimers by interaction of their C-terminal coiled-coil domains. Their N-terminal proline-rich regions interact with the SH3 domain of Rvs167. The BAR domain of Rvs167 remains free for dimerization with another N-BAR protein. (**b**) Immunoprecipitation experiments were performed on total extracts of log-phase *rvs167Δrvs161Δ* cells co-expressing Gyp5-Myc, Gyl1-HA, GFP-Rvs167 and VSV-Rvs161. Membranes were cut at the appropriate sizes for incubation with anti-Myc, anti GFP and anti-VSV antibodies. Parts of the film were grouped. The full length film is available in Supplementary Fig. S6. The image shown is representative of three independent experiments. (**c**) *rvs167Δrvs161Δ* cells co-expressing Gyp5-Myc, Gyl1-HA, GFP-Rvs167 and VSV-Rvs161 (strain OC 308, as in **b**) were synchronized by α-factor, harvested when the % of small buds reached 80%, and used for co-immunoprecipitation experiments. Membranes were cut at the appropriate sizes for incubation with anti-Myc, anti GFP and anti-VSV antibodies. Parts of the film were grouped. The full length film is available in Supplementary Fig. S6. The image shown is representative of three independent experiments.

In budding yeast, two N-BAR domain proteins were initially identified: the Rvs167 protein and its paralog Rvs161^7,8^. Both possess an N-terminal amphipathic helix, but their overall structure is different: Rvs167 contains an N-terminal BAR domain and a C-terminal SH3 domain, separated by an unstructured region, rich in glycine, proline and alanine (GPA) (Fig. 1a); Rvs161 contains only a BAR domain. The two proteins are functionally linked because the amount of Rvs167 is significantly reduced in *rvs161Δ* cells and conversely^9^. Yeast *rvs* mutants display numerous defects, including reduced viability upon starvation, sensitivity to high salt and cytotoxic compounds, defects in actin polarization, defects in endocytosis and random budding of diploid cells^10^.

Rvs167 and Rvs161 can bind and tubulate membranes *in vitro*^11^. One of their best understood functions is endocytosis: *rvs161Δ* and *rvs167Δ* are defective for α-factor internalization^8^. Rvs167 and Rvs161 associate with the endocytic vesicle neck and promote its scission from the plasma membrane after actin-driven invagination^12^. The dynamics of the recruitment of Rvs161 and Rvs167 molecules and their relationships with the other actors of endocytosis have been precisely described at the scale of the endocytic vesicle, by combinations of live-cell imaging, correlative light and electron microscopy and high throughput superresolution imaging^12–16^.

Gvp36 was identified as another BAR protein in yeast^17^. *gvp36Δ* cells share several, but not all, phenotypes with *rvs167Δ* cells, so that it was proposed that Gvp36 shares functions with Rvs167. However, there has still been no demonstration of a physical interaction between Gvp36 and either Rvs167 or Rvs161.

Rvs167 interacts with the RabGAP proteins Gyp5 and Gyl1^18–20^, two paralogs involved in the control of exocytosis, specifically at the small-bud stage^21^. The formation of a new bud in *Saccharomyces cerevisiae* involves several steps (for a review, see^22^). After the bud site selection by heritable landmarks, a local accumulation of active Cdc42-GTP recruits the formin Bni1 which nucleates actin cables oriented to the bud tip. During the initial, polarized phase of bud growth, actin cables allow the convergence of secretory vesicles to the bud tip for membrane and cargo delivery. The docking of secretory vesicles is regulated by the Rab GTPase Sec4, the major regulator of exocytosis, which ensures the recruitment of Exocyst subunits on the vesicle. Gyp5 and Gyl1 interact with Sec4 and colocalize with Sec4 at the tip of small buds^21,23^. Gyp5 acts as RabGAP protein towards Sec4^19,24^ and we have shown that Gyp5 and Gyl1 contribute to the regulation of exocytosis specifically at the stage of polarized growth of the bud^21^.

At the tip of small buds, Gyp5 and Gyl1 recruit Rvs167 for a role in exocytosis, through interaction between their N-terminal proline-rich regions and the SH3 domain of Rvs167^20^ (Fig. 1a). In this work, we attempted to identify the N-BAR protein(s) interacting with Rvs167 when recruited to the small-bud tip by Gyp5 and Gyl1. We found that Rvs161 is poorly recruited to the tip of small buds and is not co-immunoprecipitated with Gyp5 and Gyl1 in cultures enriched in small-budded cells. Co-immunoprecipitation and Bi-molecular Fluorescence Complementation (BiFC) experiments indicate that both Rvs167 and Rvs161 interact with Gvp36, and that pairs of molecules of Rvs167 interact in the absence of Rvs161 and Gvp36. BiFC experiments showed that both Rvs167/Rvs167 and Rvs167/Gyp5 interactions predominate over other combinations at the small-bud tips in living cells, suggesting that the N-BAR lattices formed at these sites are enriched in Rvs167 molecules.

Using the combination of BiFC with mRFP markers specific for different organelles, we then systematically analysed in living cells the localizations of the BiFC signals generated by combinations of the three N-BAR proteins. We show that interactions between Rvs167, Rvs161 and Gvp36 were clustered at endocytic sites at the plasma membrane, as expected, but also on the endoplasmic reticulum (ER). Importantly, the proportion of each pairing of N-BAR proteins differed between organelles. These observations imply that the composition of N-BAR protein lattices *in vivo* differs between sites. We conclude that Rvs167, Rvs161 and Gvp36 *in vivo* probably form various types of dimers and lattices of heterogeneous composition.

## Results

### Rvs161 does not co-immunoprecipitate from small-budded cells with Gyp5 and Gyl1

The interactions of Rvs167 with Gyp5 and Gyl1 involve its SH3 domain and the proline-rich regions of Gyp5 and Gyl1^20^. The BAR domain of Rvs167 is not involved in these interactions (Fig. 1a), and should therefore be free to interact with another BAR domain. We investigated the possible dimerization partners of Rvs167 in Gyp5/Gyl1/Rvs167 complexes. Rvs161 is a known dimerization partner of Rvs167, so we first examined its association, if any, with Gyp5/Gyl1/Rvs167 complexes.

Indeed, Gyp5-Myc and Gyl1-HA co-immunoprecipitated VSV-Rvs161, and VSV-Rvs161 co-precipitated Gyp5 from preparations of log-phase *rvs167Δrvs161Δ* cells co-expressing Gyp5-Myc, Gyl1-HA, GFP-Rvs167 and VSV-Rvs161 (Fig. 1b). This indicates that Gyp5/Gyl1/Rvs167 complexes formed *in vivo* in exponentially growing cells recruit Rvs161 molecules.

We then examined these interactions in small-budded cells. We performed further co-immunoprecipitation experiments with *rvs167Δrvs161Δ* cells co-expressing Gyp5-Myc, GFP-Rvs167 and VSV-Rvs161, synchronized by α-factor and harvested at the small-bud stage (Fig. 1c). VSV-Rvs161 strongly co-immunoprecipitated GFP-Rvs167 and *vice versa*, indicating that the two proteins interact at this stage of the cell-cycle. Gyp5 co-immunoprecipitated a fraction of Rvs167 (approximately 5% of the total Rvs167 as previously described^20^) but did not co-immunoprecipitate Rvs161. Conversely, VSV-Rvs161 did not co-immunoprecipitate Gyp5. Therefore, Rvs161 is absent from the Gyp5/Gyl1/Rvs167 complexes formed in small-budded cells, or present in amounts that our co-immunoprecipitation experiments were unable to detect.

N-BAR proteins form dimers and lattices when associated with membranes, and consequently Rvs167 may interact with N-BAR proteins others than Rvs161.

### Both Rvs167 and Rvs161 interact *in vivo* with the N-BAR protein Gvp36

We tested for interactions between Rvs167 and another BAR protein found in yeast, Gvp36^17^. A *gvp36Δ* mutant shares several phenotypes with a *rvs167Δ* mutant, consistent with Gvp36 and Rvs167 cooperating in some cellular functions. The BAR domain of Gvp36 is similar to that of Rvs167 and Rvs161, according to the Constraint-based Multiple Protein Alignment Tool^25^ and to the classification of the Conserved Domains Database^26^. Heliquest software^27^ predicts that the 18 N-terminal residues of Gvp36 form an amphipathic helix, like the N-terminal residues of Rvs167 and Rvs161. Therefore, Gvp36 is a member of the family of N-BAR proteins^3^.

**Figure 2:**
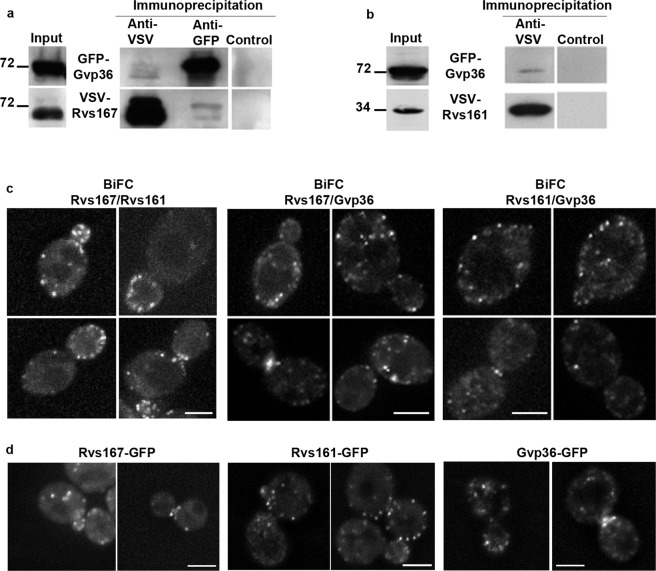
Both Rvs167 and Rvs161 interact with the N-BAR protein Gvp36. **(a**) Immunoprecipitation experiments were performed with total extracts of log-phase *rvs167Δgvp36Δ* cells co-expressing GFP-Gvp36 and VSV-Rvs167 fusion proteins. Membranes were cut at the appropriate sizes for incubation with anti GFP and anti-VSV antibodies. Parts of the film were grouped. The full length film is available in Supplementary Fig. S6. The image shown is representative of three independent experiments. (**b**) Immunoprecipitation experiments were performed with total extracts of log-phase *rvs161Δgvp36Δ* cells co-expressing GFP-Gvp36 and VSV-Rvs161 fusion proteins. Membranes were cut at the appropriate sizes for incubation with anti GFP and anti-VSV antibodies. Parts of the film were grouped. The full length film is available in Supplementary Fig. S6. The image shown is representative of two independent experiments. (**c**) Interaction of both Rvs167 and Rvs161 with Gvp36 generate BiFC signals. Representative images of the BiFC signals generated in cells co-expressing the indicated pairs of N-BAR proteins at various stages of the cell cycle. The scale bar, 3 µm, is applicable to all the cells of a square. (**d**) Representative images of cells expressing the fusion proteins Rvs167-GFP, Rvs161-GFP or Gvp36-GFP as indicated. The scale bar, 3 µm, is applicable to all the cells of a square.

Small amounts of Gvp36 and Rvs167 co-immunoprecipitated from log-phase *rvs167Δgvp36Δ* cells co-expressing GFP-Gvp36 and VSV-Rvs167 (Fig. 2a). Similarly, Rvs161 co-immunoprecipitated small amounts of Gvp36 in *rvs161Δgvp36Δ* cells co-expressing GFP-Gvp36 and VSV-Rvs161 (Fig. 2b). These results indicate that both Rvs167 and Rvs161 forms complexes *in vivo* with Gvp36, although the abundance of these complexes in cells appears to be low.

We also used BiFC assays^28^ to detect interactions between Rvs167, Rvs161 and Gvp36. Briefly, we obtained strains expressing Rvs167, Rvs161 or Gvp36 fused at their C-terminus with either the N-terminal part (VN) or the C-terminal part (VC) of the Venus YFP; we crossed these strains and looked in living diploid cells for YFP signals indicating interactions of the two fusion proteins (Supplementary Fig. S1a). No significant signal was detected in haploid cells harboring only -VN or -VC fusion proteins, or in diploid cells harboring proteins which do not interact with Rvs167 or Rvs161 (endo-β1,3-glucanase Bgl2 and the nucleoporin Nic96 were used as negative controls, Fig. S1b). One further control was added: co-immunoprecipitation experiments in diploid cells co-expressing Gyl1-Myc and Gyl1-HA show that Gyl1-Myc does not co-immunoprecipitate with Gyl1-HA, and conversely (Fig. S1c). This indicates that the two tagged forms of Gyl1 do not associate in same complexes and excludes, in particular, the existence of Gyl1-Gyl1 homodimers. We scored small buds (defined by the ratio (R) of bud width to bud neck width ≤ 1) for BiFC signal in cells co-expressing either Gyp5-VN/Gyl1-VC or Gyl1-VN/Gyl1-VC. As shown in Fig. S1d, no BiFC signal was found in the small buds of cells co-expressing Gyl1-VN/Gyl1-VC. This result indicates that the proximity of -VN and -VC fusion proteins is not sufficient by itself to generate BiFC signal, even when these molecules are concentrated in the restreint space of the small bud.

We tested the interactions of each pair of N-BAR proteins in diploid cells in the two possible combinations (e.g. Rvs167-VN/Gvp36-VC and Rvs167-VC/Gvp36-VN), by crossing haploid strains expressing Rvs167, Rvs161 and Gvp36 –VN or –VC fusion proteins. In general, both combinations gave similar results, so they are not presented separately below. Significant YFP signals were detected in living cells for the pair Rvs167/Rvs161; weaker but significant signals were detected for the pairs Rvs167/Gvp36 and Rvs161/Gvp36 (Fig. 2c). These BiFC signals were similar to those generated by Rvs167-, Rvs161- or Gvp36-GFP fusion proteins (Fig. 2d).

Therefore, both immunoprecipitation and BiFC experiments provide evidence that Rvs167 and Rvs161 interact *in vivo* with Gvp36 in exponentially growing cells.

### Rvs167 molecules can form complexes *in vivo* independently of Rvs161 and Gvp36

**Figure 3:**
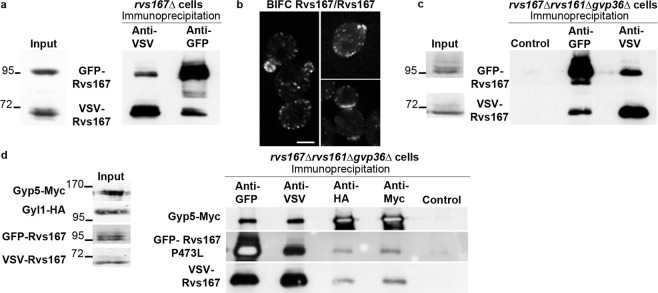
Rvs167 molecules interact independently of Rvs161 and Gvp36. (**a**) Immunoprecipitation experiments were performed with total extracts of log-phase *rvs167Δ* cells co-expressing GFP-Rvs167 and VSV-Rvs167 fusion proteins. Membranes were cut at the appropriate sizes for incubation with anti-GFP and anti-VSV antibodies. Parts of the film were grouped. The full length film is available in Supplementary Fig. S6. The image shown is representative of three independent experiments. (**b**) Representative images of the BiFC signals generated in cells co-expressing Rvs167-VN and Rvs167-VC at various stages of the cell cycle. The scale bar, 3 µm, is applicable to all the cells of the square. (**c)** Immunoprecipitation experiments were performed with total extracts of log-phase *rvs167Δrvs161Δgvp36Δ* cells co-expressing GFP-Rvs167 and VSV-Rvs167 fusion proteins. The image shown is representative of three independent experiments. (**d**) Immunoprecipitation experiments were performed with total extracts of log-phase *rvs167Δrvs161Δgvp36Δ* cells co-expressing GFP-Rvs167 P473L, VSV-Rvs167, Gyp5-Myc and Gyl1-HA fusion proteins. Membranes were cut at the appropriate sizes for incubation with anti-Myc, anti-GFP and anti-VSV antibodies. Parts of the film were grouped. The full length film is available in Supplementary Fig. S6. The image shown is representative of three independent experiments.

We then examined the possibility of homodimerization of Rvs167. From log-phase *rvs167Δ* cells co-expressing GFP-Rvs167 and VSV-Rvs167, the fusion proteins are efficiently co-immunoprecipitated, indicating that Rvs167 forms complexes *in vivo* including at least two Rvs167 molecules (Fig. 3a). Consistent with this, significant BiFC signals were observed in diploid cells co-expressing the Rvs167-VN and Rvs167-VC fusion proteins (Fig. 3b).

The interaction between Rvs167 molecules has been observed previously by BiFC^11^. However, because N-BAR proteins are able to form lattices, Youn *et al*. suggested that the BiFC signals observed in strains co-expressing Rvs167-VN and Rvs167-VC were the result of lattice formation: with lattices composed of Rvs167/Rvs161 dimers, molecules of Rvs167 may be close enough to allow the reconstitution of a Venus YFP molecule, generating a BiFC signal. If true, this hypothesis would also explain the results obtained in our co-immunoprecipitation experiment, if the immunoprecipitates contained GFP-Rvs167 and VSV-Rvs167 molecules interacting *via* Rvs161 molecules, for example.

To confirm or disprove this possibility, we repeated the co-immunoprecipitation experiments with *rvs167Δrvs161Δgvp36Δ* cells (Fig. 3c). GFP-Rvs167 and VSV-Rvs167 co-immunoprecipitated, and thus formed complexes, as efficiently in the presence (Fig. 3a) and absence (Fig. 3c) of Rvs161 and Gvp36. We also exploited the P473L mutation in the SH3 domain of Rvs167, which impairs the interaction of Rvs167 with Gyp5 and Gyl1 *in vitro* and *in vivo*^19,20,29^. Co-expressed in *rvs167Δrvs161Δgvp36Δ* cells, the mutant protein GFP-Rvs167 P473L and the native protein VSV-Rvs167 co-immunoprecipitated efficiently (Fig. 3d), indicating that the native and mutant forms of Rvs167 are able to form complexes. The relative amounts of the native and mutant forms of Rvs167 co-immunoprecipitated with Gyp5-Myc or Gyl1-HA were similar. The SH3 domain of the P473L mutant is unable to mediate interactions with Gyp5 and Gyl1. Therefore, Rvs167-P473L appears to associate with the immunoprecipitated complex *via* its BAR domain dimerizing with the BAR domain of VSV-Rvs167. These various findings indicate that Rvs167 molecules can interact *in vivo* in the absence of Rvs161 and Gvp36, and therefore that they interact together directly, probably via their BAR domain.

However, we cannot absolutely exclude that Rvs167 could form dimers with still unknown partners. Moreover, the ability of N-BAR proteins to form lattices makes it difficult to assert from these results that the interaction of Rvs167 molecules is direct, even in the absence of Rvs161 and Gvp36. We further reasoned that if Rvs167 molecules were incorporated into lattices uniformly composed, for instance, of regularly distributed Rvs167/Rvs161 dimers, then Rvs167/Rvs161 and Rvs167/Rvs167 BiFC signals should display the same distribution in cells. We therefore compared the distribution in living cells of the BiFC signals generated by each pair of N-BAR proteins.

### Rvs167/Rvs167 interactions and Rvs167/Gyp5 interactions predominate at the tip of small buds

First, we investigated the BiFC signals at the particular site of the small-bud tip, because we previously demonstrated that Rvs167 is recruited to the tip of small buds (ratio R of bud width to bud neck width ≤1) by an interaction with Gyp5 and Gyl1^20^.

**Figure 4:**
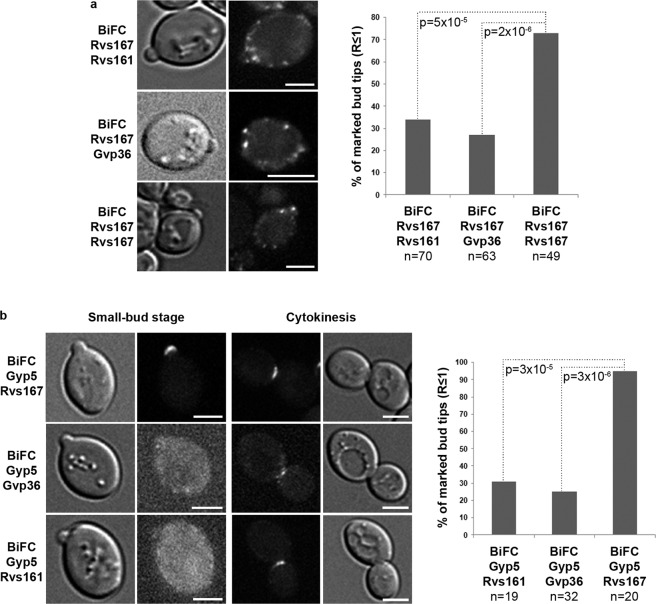
BiFC reveals different distributions and interactions for the three pairs of N-BAR proteins at the tip of small buds. (**a**) Small bud tips were scored for BiFC signals corresponding to each indicated pair of N-BAR proteins. Representative images of small buds are shown on the left. Scale bar, 3 µm. n is the number of small bud tips scored. p values indicate the probability for identity between observed distributions (Chi^2^ test). (**b**) Small bud tips were scored for BiFC signals corresponding to the interaction of Gyp5 with each indicated N-BAR protein. Representative images of small buds are shown on the left. Representative images of cytokinesis stages in each strain are shown as positive controls. Scale bar, 3 µm. n is the number of small bud tips scored. p values indicate the probability for identity between observed distributions (Chi^2^ test).

We scored tips of small buds (R ≤ 1) for the presence of BiFC signals in strains expressing three different pairs of fusion proteins (Fig. 4a). A BiFC signal was found at the tip of 73% of the small buds of cells expressing the Rvs167/Rvs167 pair. BiFC signals were significantly less frequent at small-bud tips in cells expressing the Rvs167/Rvs161 and Rvs167/Gvp36 pairs. This suggests that the N-BAR lattices at small-bud tips are enriched in Rvs167 molecules.

We also investigated the interaction of Gyp5 with each N-BAR protein in small buds of living cells (Fig. 4b). A strong BiFC signal was observed at the tip of 95% of the small buds for the pair Gyp5/Rvs167, confirming that Gyp5 interacts with Rvs167 at the bud tip. By contrast, signals were significantly less frequent and also extremely weak for the Gyp5/Gvp36 and Gyp5/Rvs161 pairs. As a control, there were significant signals at the bud neck during cytokinesis (Fig. 4b, right panel) confirming that the BiFC technique effectively reveals any interactions between Gyp5 and Rvs161 or Gvp36. These findings indicate that Gyp5 interacts mostly with Rvs167 at the tip of small buds, consistent with lattices at these sites being Rvs167-enriched.

Two other results support these data (Supplementary Fig. S2). First, we determined the localization of Rvs161-GFP at the tip of buds of various sizes in living cells, as previously described for Rvs167-GFP (^20^ and see Methods). Rvs161-GFP marked only 20% of the small-buds (R ≤ 1), whereas >80% of small-budded cells displayed a patch of Rvs167 at the bud tip (Supplementary Fig. S2a). This result is in agreement with the frequency of BiFC signal found for the pair Rvs167/Rvs161 in Fig. 4a.

Second, we assessed the involvement of Rvs161 and Gvp36 in secretion at the small-bud stage. We have shown previously that the secretion of the β-1,3-endoglucanase Bgl2 is defective at 13 °C in cultures of *rvs167Δ* strains enriched in small-budded cells^20^. We conducted similar experiments in *rvs161Δ* and *gvp36Δ* strains. Briefly, cells expressing Bgl2-HA under the control of the GAL promoter were arrested in G1 by glucose starvation, then shifted to 2% galactose to initiate a new cell cycle. Cells were harvested at the small-budded stage, and internal and secreted Bgl2-HA were quantified to determine the percentage of Bgl2 secreted. No significant defect was found either in *rvs161Δ* or *gvp36Δ* strains, at time-points when *rvs167Δ* cells displayed a significant decrease in Bgl2-HA secretion (Supplementary Fig. S2b). This indicates that, unlike Rvs167, Rvs161 and Gvp36 are not involved in the control of secretion at the small-bud stage.

These results indicate that the N-BAR lattices formed at the tip of small buds are enriched in Rvs167 molecules, which interact with Gyp5 and are involved in secretion at the small-bud stage independently of Rvs161 and Gvp36.

It is therefore possible that N-BAR proteins form lattices of variable composition *in vivo*. This led us to investigate in more detail the distribution of the BiFC signals generated in living cells by each pair of N-BAR proteins.

### The distribution of Rvs167, Rvs161 and Gvp36 interactions differs between organelles in living cells

We obtained strains co-expressing the –VN and –VC fusion proteins with a collection of mRFP markers specific for different organelles^30^. Exponentially growing cells were examined for YFP and mRFP signals and colocalization events were quantified plane-to-plane in each cell (see Methods for details).

**Figure 5:**
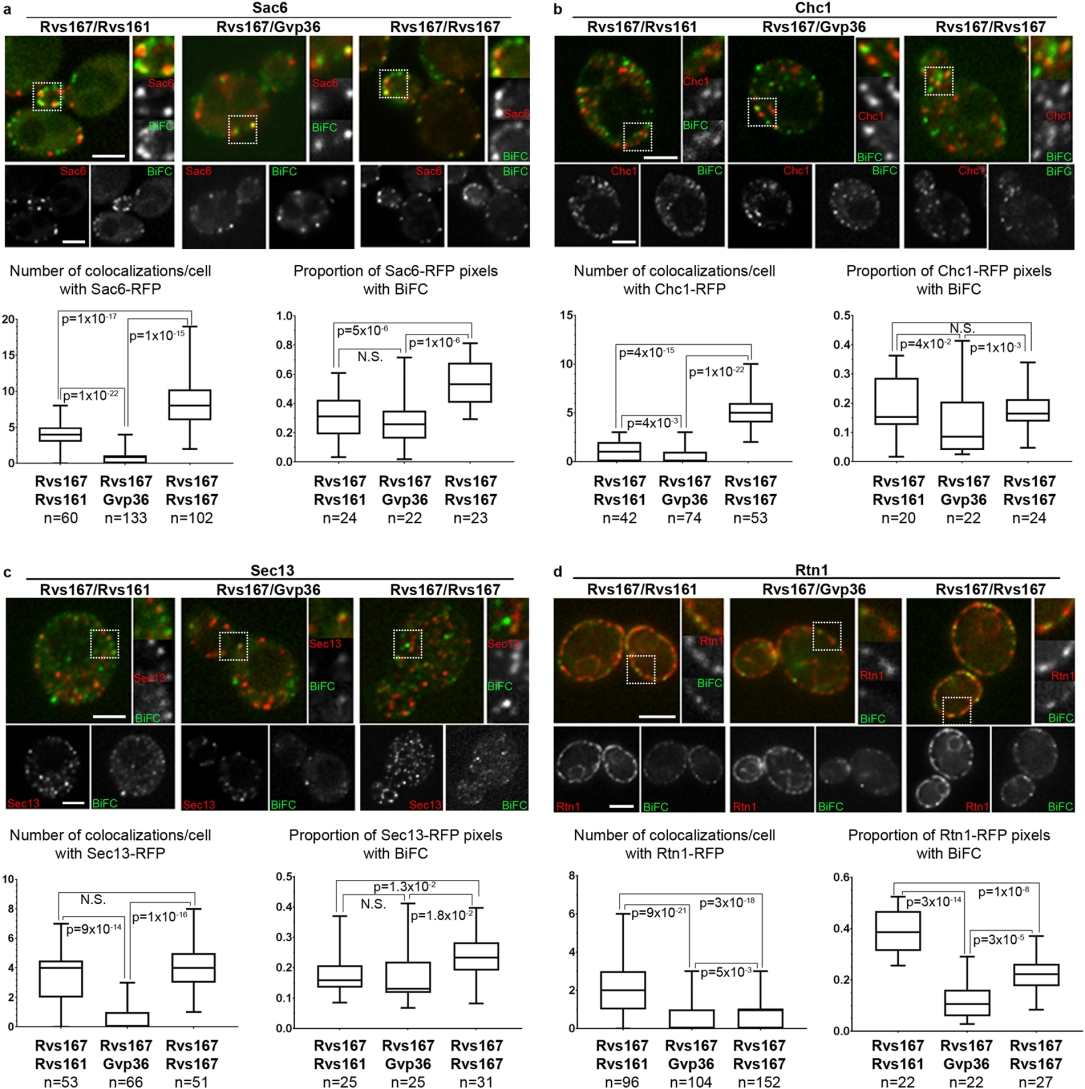
Colocalization of the BiFC signals generated by pairs of N-BAR proteins with Sac6, Chc1, Sec13 and Rtn1. Representative images of colocalization events in cells co-expressing each pair of N-BAR proteins and the indicated mRFP marker. One single plane from a z-stack is shown. The scale bar, 3 µm, is applicable to all the cells of the panel. For each panel, quantifications and statistical analysis of the results are shown. Left: number of colocalizations/cell with each marker. Right: proportion of mRFP positive pixels containing BiFC signal (Manders M2 coefficient). Detailed quantification of the colocalization events are shown in Supplementary Table S2. The box plots indicate the mean ± S.D., minimal and maximal values. n is the number of cells scored. Statistical differences between the observed distributions were tested by the Wilcoxon rank sum test. The indicated p values represent the probability of identity of the two distributions. N.S. indicates a p value > 0.05.

Figure 5 shows representative images of colocalization events with four mRFP markers for the three pairs of N-BAR proteins and a quantitative representation of the colocalization events per cell (detailed quantitative data are presented in Supplementary Table S2). To further quantify the colocalization events, the proportion of mRFP positive pixels containing BiFC signal (Manders M2 coefficient) was determined for a panel of at least 20 cells in each category. The distributions of M2 coefficient values is shown.

**Figure 6:**
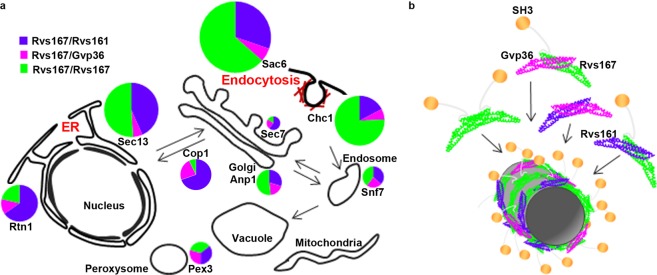
The relative proportions of interactions of three pairs of N-BAR proteins, as revealed by BiFC, vary between sites. (**a**) The subcellular distribution of the interactions between three N-BAR proteins *in vivo* as assessed by BiFC, established from the quantitative results shown in Supplementary Table S2. For each mRFP marker, the area of the circle is proportional to the sum of the arbitrary values found, thus reflecting the abundance of colocalized interactions; the sectors indicate the relative contribution of each pair of N-BAR proteins. No significant BiFC signals were found in the nucleus (both DAPI staining and the nucleoporin Nic96-mRFP were tested), vacuoles (stained with FM4-64), or mitochondria (stained with DAPI or mt-RFP). (**b**) A speculative model of the formation of heterogeneous N-BAR lattices, showing incorporation of various types of N-BAR protein dimers in a lattice.

All colocalizations of BiFC signals with mRFP markers were partial colocalizations, indicating that the three N-BAR proteins are more or less associated with the membranes of diverse organelles. The largest numbers of colocalization events per cell were found with four specific markers: the yeast fimbrin Sac6, the clathrin heavy chain Chc1 and the endoplasmic reticulum (ER) markers Sec13 and Rtn1. The colocalization with Sac6 is in agreement with the known localizations of Rvs167 and Rvs161 at the endocytic sites^12,31,32^. Chc1 is involved in endocytosis, but also in Golgi to endosomes transport^33^; however, we noticed first that most of the colocalization events located just below the plasma membrane and second that colocalization events of BiFC signals with both Golgi and endosomes were very rare (see Fig. 6a and Supplementary Table 2), two elements strongly suggesting that most of the colocalization events of the BiFC signals with Chc1 correspond to endocytic sites. Sec13 belongs to the core components of the COPII vesicle coat required for ER to Golgi transport^34^ and Rtn1 is a reticulon protein located to the peripheral tubular ER^35^.

Importantly, the number of colocalization events per cell was systematically different for each pair of N-BAR proteins tested. For instance, a mean of 8 colocalization events per cell were found for the Rvs167/Rvs167 BiFC signals with the actin-filaments-crossing protein Sac6, versus only 4 colocalization events per cell for Rvs167/Rvs161 and 1.5 for Rvs167/Gvp36 (Fig. 5a and Supplementary Table S2). Statistical analyses indicated that the distributions of the numbers of colocalization events with Sac6, as well as the distributions of the M2 coefficent values, differ significantly between the three pairs of N-BAR proteins (Fig. 5a). This was also the case for the colocalizations with the specific markers Chc1, Sec13 and Rtn1 (Fig. 5b–d and Supplementary Table S2).

BiFC signals also colocalized with the ER marker Cop1, the endosomal marker Snf7, the Golgi apparatus markers Anp1 and Sec7 and the peroxysomal marker Pex3. Representative images of colocalization events with these markers for the three pairs of N-BAR proteins and a quantitative representation of the colocalization events per cell are shown in Supplementary Fig. S3. Statistical analyses indicated that the distributions of the number of colocalization events with Cop1, Anp1 and Sec7 differ significantly between the three pairs of N-BAR proteins. No significant BiFC signals were found in the nucleus (both DAPI staining and the nucleoporin Nic96-mRFP were tested), vacuoles (stained with FM4-64), or mitochondria (stained with DAPI or mt-RFP).

A summary of the colocalization events found in these experiments, illustrating both the abundance of colocalized interactions and the relative contribution of each pair of N-BAR proteins, is shown in Fig. 6a. It underlines two conclusions of these results:

- The first one is that endocytic sites, as already known, but also the ER are the main membrane sites where the three N-BAR proteins establish interactions *in vivo*;
- The second, and the most important, is that the proportion of interactions between each pair of N-BAR proteins differs between cell sites. For instance, Rvs167/Rvs167 interactions predominate at endocytic sites whereas Rvs167/Rvs161 interactions are more abundant at the ER. Such differences would not be found if the lattices formed had the same composition from site to site. Therefore, this strongly suggests that the lattices formed on each organelle are not of uniform composition, but rather incorporate different proportions of the various N-BAR proteins.

### The absence of the Rvs161 protein does not preclude the formation of lattices enriched in Rvs167 molecules

We wondered whether a modification of the expression level of Rvs161 or Gvp36 would

influence the localization of Rvs167/Rvs167 interactions, or Rvs167 functions.

Cells were transformed with plasmids overexpressing VSV-Rvs161, GFP-Gvp36, or both (Supplementary Fig. S5a). In cells overexpressing VSV-Rvs161, the colocalization of Rvs167/Rvs167 BiFC signal with Cop1, Chc1 and Sac6 was not significantly modified (Fig. S5b). The function of Rvs167 in budding polarity was assessed by calcofluor staining of bud scars of diploid cells^36^. Figure S5c shows that the budding polarity was not significantly altered in diploid cells overexpressing VSV-Rvs61, GFP-Gvp36, or both. A mild, but reproducible, growth defect on 6% NaCl was observed for haploid cells overexpressing both VSV-Rvs161 and GFP-Gvp36 (Fig. S5d). This indicates that, at least in our conditions, Rvs167 localizations and functions are insensitive to overexpression of other N-BAR proteins.

**Figure 7:**
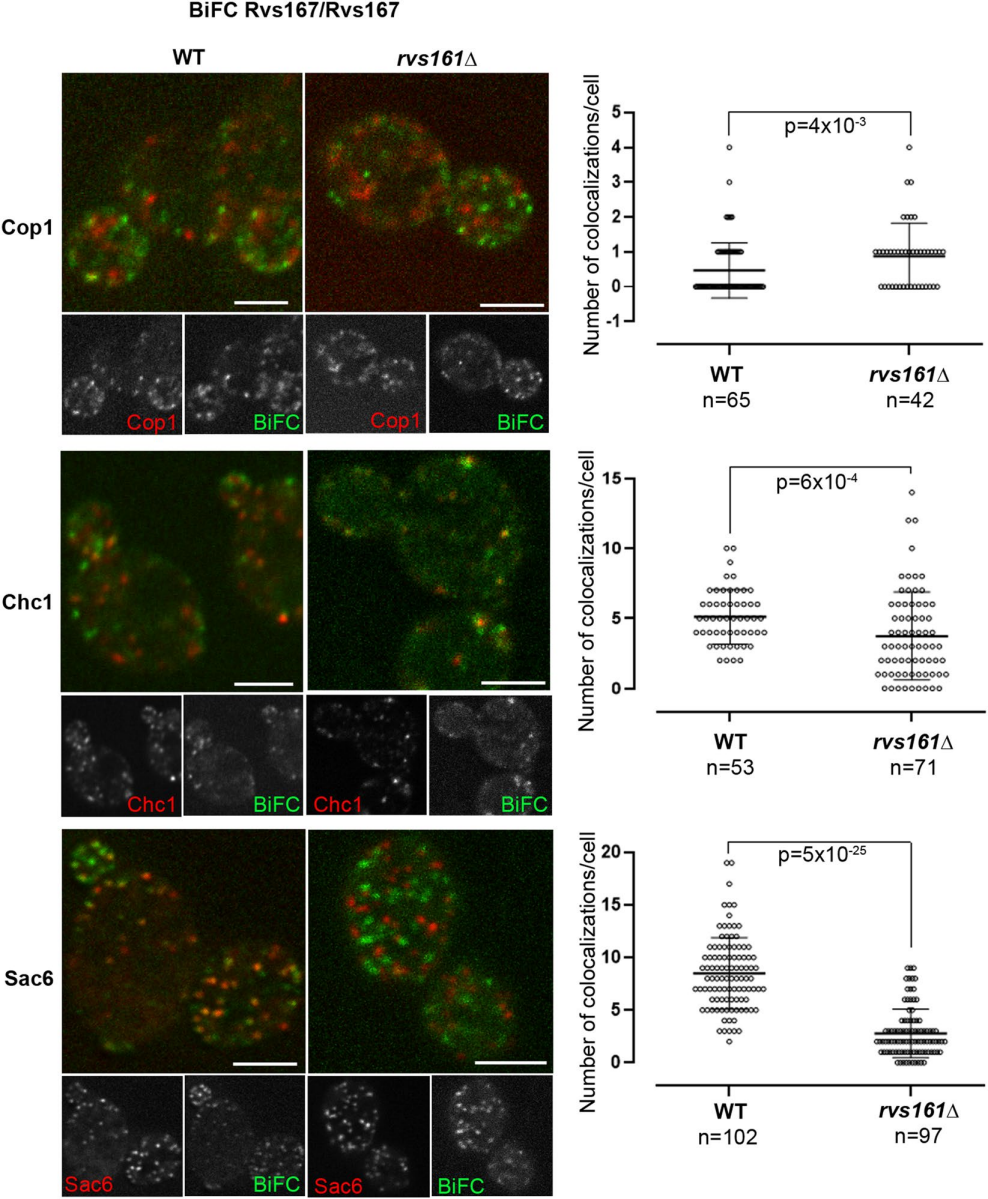
Rvs167/Rvs167 lattices form in *rvs161Δ* cells and their localization is modified. Representative images of BiFC signal in WT or *rvs161Δ* cells co-expressing the Rvs167-VN and Rvs167-VC fusion proteins and the indicated mRFP marker. One single plane from a z-stack is shown. Scale bar, 3 µm. For each panel, the number of colocalizations/cell a and statistical analysis of the results are shown. The box plots indicate the mean ± S.D., minimal and maximal values. n is the number of cells scored. Statistical differences between the observed distributions were tested by the Wilcoxon rank sum test. The indicated p values represent the probability of identity of the two distributions.

We also examined by BiFC whether Rvs167/Rvs167 interactions are disrupted in *rvs161Δ* cells. Importantly, a punctate Rvs167/Rvs167 BiFC signal was detected in *rvs161Δ* cells as in wild-type cells (Fig. 7), except that the signal was weaker. This result confirms that Rvs167 molecules can form complexes *in vivo* independently of Rvs161, as shown above by our co-immunoprecipitation experiments.

The colocalization of Rvs167/Rvs167 BiFC signal with different mRFP markers was scored. The number of colocalizations per cell with the ER marker Cop1 was only moderately increased. Noticeably, the number of colocalizations per cell with Chc1 decreased also moderately whereas the number of colocalizations with Sac6 decreased more strongly. Although both Chc1 and Sac6 are involved in the formation of endocytosis vesicles, it was shown by precise analysis of endocytosis dynamics^12,14–16^ that both the time and the place of their recruitment are different: clathrin molecules are recruited to the plasma membrane at the very beginning of the endocytic vesicle formation and remain associated with it until scission; Sac6, an actin crosslinker, is recruited during the formation of the actin network around the membrane invagination^14,15^. Moreover, it was shown that the dynamics of endocytosis vesicles is disrupted in *rvs161Δ* cells, with approximately one third of the vesicles retracting toward the cell surface after their formation^12^. Our results indicate that lattices enriched in Rvs167 molecules can form at endocytic sites, near the clathrin molecules, independently of Rvs161. Subsequently, the absence of Rvs161 could lead to destabilization of the lattices or disruption of the dynamics of endocytosis, explaining the decrease of colocalizations with Sac6 that we observe.

## Discussion

It was known that the yeast *Candida albicans* possesses several N-BAR proteins, which form *in vivo* at least two forms of heterodimers^37^. In budding yeast also, in which Rvs167 and Rvs161 were known since 1993, recent reports had suggested that the N-BAR proteins landscape is actually complex. A new N-BAR protein, Gvp36, was identified^17^. It was shown that, in mating cells, Rvs161 interacts independently of Rvs167 with the pheromone-induced protein Fus2, which harbors an N-BAR domain^38,39^. Also, Rvs167 was shown to interact, independently of Rvs161, with components of the SAGA complex in the nucleus^40^.

Here, we perform an extensive study of the interactions of Rvs167 with two other N-BAR proteins in living cells, Rvs161 and Gvp36. Starting from a particular situation where Rvs167 is recruited to the tip of small buds by the RabGAP proteins Gyp5 and Gyl1, we show that BiFC signals corresponding to Rvs167/Rvs161 and Rvs167/Gvp36 interactions are much less frequent at the tip of small buds than those generated by Rvs167/Rvs167 interactions. Accordingly, BiFC signals corresponding to Gyp5/Rvs161 and Gyp5/Gvp36 interactions are much less frequent than those resulting from Gyp5/Rvs167 interactions. These results indicate that lattices formed at the particular site of small buds are enriched in Rvs167 molecules.

Discrepancy of this type between the BiFC signals generated by the three pairs of N-BAR proteins was found for almost all organelles or cell sites where they localize: the numbers of BiFC signals colocalizing with a particular organelle varied, in most cases with high statistical significance. Assuming that the frequency of the BiFC signals reflects the frequency of interactions within lattices, this is strong evidence that the three N-BAR proteins form lattices of different compositions at different sites. Thus, our results provide an insight into complexity of N-BAR protein lattices *in vivo*. Figure 6b presents a very simple speculative model for the formation of lattices of heterogeneous composition, in which the proportion of several heterodimers assembling into lattices varies. Although not represented, the formation of Rvs161 or Gvp36 homodimers is also a possibility, thus generating a number of possible combinations and possibilities of fine variations in the lattice composition, according to place and time. This model is in agreement with the fact that we did not identify, on any organelle, lattices exclusively formed of one N-BAR protein or even one pair of N-BAR proteins. Moreover it is in agreement with the fact that the cellular functions of these three N-BAR proteins do overlap but are not identical, as shown by the defects found in functional analysis of mutant strains^10,17^.

Noticeably, we found that the proportions of Rvs167/Rvs161 and Rvs167/Rvs167 interactions differ significantly between the plasma membrane and ER: Rvs167/Rvs167 interactions predominate at the plasma membrane, whereas Rvs167/Rvs161 interactions predominate at the ER membrane. A functional link of Rvs167 and Rvs161 with ER was already proposed^19^, on the basis of genetic interactions. Our results confirm that Rvs167, Rvs161 and, to a lesser extent, Gvp36 are recruited to the ER in exponentially growing cells, which makes this organelle their second major location site. However, it was demonstrated that *rvs167* and *rvs161* mutants exhibit normal ER-to-Golgi trafficking^8,10^, so that their functions in this organelle remain unknown. The colocalization of Rvs161 and Rvs167 with Rtn1 and Sec13 might be a clue for a role in peripheral ER maintenance.

We report co-immunoprecipitation and BiFC experiments showing that both Rvs167 and Rvs161 indeed interact with Gvp36 *in vivo*. However, although Gvp36 is approximately as abundant in cells than Rvs161 (>7000 molecules/cell, according to the GFP localization database^41^), we were able to immunoprecipitate only small amounts of Rvs167/Gvp36 complexes. Similarly, the BiFC signals generated by the Rvs167/Gvp36 fusion proteins were always weaker than those generated by other pairs (see Fig. 2) and fewer Rvs167/Gvp36 BiFC signals colocalized with any mRFP marker. Thus, our results suggest that Gvp36 could be a minor component of Rvs167/Rvs161/Gvp36 lattices, or may associate with lattices. One noticeable exception is the Golgi apparatus, where up to 30% of the colocalization events involved Rvs167/Gvp36 interactions. This is in agreement with the fact that Gvp36 is a peripheral membrane protein associated with the Sed5-positive early Golgi compartment^42^.

We had shown previously that small-budded *rvs167Δ* accumulate secretory vesicles and are defective for Bgl2 secretion at 13 °C^20^. However, it was demonstrated that endocytosis and exocytosis are coupled during bud growth and that endocytosis defects can affect exocytosis and polarity^43^. This suggested that the secretion defects of *rvs167Δ* small-budded cells could be a consequence of their endocytosis defect. Here, we show that neither Rvs161 nor Gvp36 are involved in Bgl2 secretion at the small-bud stage, whereas their involvment in endocytosis is well-documented. These results indicate that the secretion defects of *rvs167Δ* small-budded cells cannot be due to endocytosis defects and bring a new argument for the hypothesis of a specific role of Rvs167 in exocytosis in polarized growth of small buds.

## Methods

### Plasmids

The plasmids pGFP-Rvs167, pGFP-Rvs167p-P473L and pBgl2-HA, expressing GFP-Rvs167, GFP-Rvs167 P473L and Bgl2-HA, respectively, were described in^20,23,37^.

#### pVSV-Rvs167

The RVS167 coding sequence was amplified by PCR from genomic DNA of a BY4741/42 strain of *S. cerevisiae*, with oligonucleotides designed to insert: at the 5′ terminus, a HindIII site, a strong ATG followed by the VSV-Tag coding sequence in-frame with the RVS167 coding sequence; at the 3′ terminus, a XbaI site. The amplified fragment was inserted between the HindIII and XbaI restriction sites of the vector YCp-ADH1^44^ and verified by sequencing.

#### pVSV-Rvs161

The RVS161 coding sequence was amplified by PCR from genomic DNA of a BY4741/42 strain of *S. cerevisiae*, with oligonucleotides designed to insert: at the 5′ terminus a BamHI site, a strong ATG followed by the VSV-Tag coding sequence in-frame with the RVS161 coding sequence; at the 3′ terminus, a XbaI site. The amplified fragment was inserted between the BamHI and XbaI restriction sites of the vector YCp-ADH1 and verified by sequencing.

#### pGFP-Gvp36

The GVP36 coding sequence was amplified by PCR from genomic DNA of a BY4741/42 strain of *S. cerevisiae*, with oligonucleotides designed to eliminate the ATG and to introduce SpeI (5′) and XhoI (3′) restriction sites. The amplified fragment was inserted between the SpeI and XhoI restriction sites of pUG36 and verified by sequencing.

Expression, stability and functionality of the Rvs167-, Rvs161- and Gvp36 fusion proteins were assessed by Western blot and complementation assay (Supplemental Fig. S4).

### Media and growth conditions

Cells were harvested at an OD_600nm_ of 0.6–1.0, for all purposes. In all the experiments including strains transformed with pUG36 encoding GFP-Rvs167p or Gvp36p expressed under the control of the MET25 promoter, cells were grown in Complete Synthetic (CS) media (MP Biomedicals) containing 20 mg/L methionine to limit overproduction of the fusion proteins.

For synchronization experiments, α-factor (Sigma-Aldrich) was added to a final concentration of 10 μg/ml to cultures in early log phase, and culture was continued for three hours. The cells were pelleted by centrifugation, washed and resuspended in fresh culture medium. The percentage of small-budded cells was scored every ten minutes. Cells were harvested when the percentage of small-budded cells reached 80%. Volumes of cultures corresponding to 1.5 × 10^8^ cells were quickly frozen in liquid nitrogen for the co-immunoprecipitation experiments.

For the Bgl2p-HA secretion assays, cells in the early exponential growth phase cultured at 30 °C in CS medium containing 2% glucose were shifted to 13 °C for 24 h, then transferred to CS medium containing 0.1% glucose and cultured for 26 h at 13 °C. They were then transferred to CS medium containing 2% galactose: the OD_600nm_ was measured at the times indicated and aliquots of the cultures were incubated with 200 μg/mL Phloxin B (Sigma-Aldrich) to determine cell viability and the percentage of small-budded cells. Volumes of cultures corresponding to 3 × 10^7^ cells were quickly frozen in liquid nitrogen for Bgl2p-HA secretion assays.

### Immunoprecipitation experiments

Volumes of cell lysates corresponding to 3 × 10^8^−6 × 10^8^ cells (for Fig. 2b) were diluted in 500 µl of cell lysis buffer (20 mM Tris-HCl pH 7.5, 100 mM NaCl, 1 mM EDTA, 0.5% Triton X-100, plus 1 mM PMSF and proteases inhibitors). After a one hour incubation at 4 °C with gentle shaking, extracts were centrifuged at 15,000 × g for 5 minutes. 1 µg/10^7^ cells of antibodies was added to supernatants. The antibodies used for immunoprecipitation were mouse monoclonal anti-Myc clone 9E10, rat monoclonal anti-HA clone 3F10, mouse monoclonal anti-GFP clones 7.1 and 13.1, mouse monoclonal anti-VSV clone P5D4 (all from Roche). Protein G-agarose beads (240 µl of a 50% slurry, prewashed in lysis buffer) were added and the mixture was incubated for 2 hours at 4 °C with gentle shaking. The beads were washed twice with lysis buffer, twice with 100 mM Tris-HCl pH7.5, 300 mM NaCl, and once with 20 mM Tris-HCl pH7.5. Beads were then resuspended in 25 µl of Laemmli sample buffer, boiled for 5 minutes, and centrifuged for 15 minutes at 20,000 × g. For negative controls, co-immunoprecipitations were performed exactly as described, except that antibodies were omitted. The resulting supernatant was submitted to SDS-PAGE. Membranes were cut at the appropriate sizes for incubation with the indicated antibodies. Revelation was performed by chimioluminescence. The inputs correspond to the total protein extracts of 3 × 10^7^ cells. In some cases (Figs. 1b and 3c,d) revelation was performed with alkaline phosphatase for a better resolution of the signals.

### Construction of BiFC strains

Strains used in this study are listed in Supplementary Table S1. BiFC strains were obtained exactly as described in^28^, using the set of BiFC plasmids supplied by Bioneer. For the colocalization experiments, strains harboring Rvs167, Rvs161 or Gvp36 fused to the Venus N-terminal moiety were crossed to a collection of strains harboring proteins fused to mRFP^30^ and the resulting strains were sporulated. His^+^ Kan^R^ cells were selected and crossed to the strains harboring Rvs167, Rvs161 or Gvp36 fused to the C-terminal moiety.

### Light microscopy and scoring

Cells were observed in a three-dimensional deconvolution microscope DMIRE2 (Leica Microsystems) equipped with an HCxPL APO 100 × oil CS objective, NA = 1.40 (Leica Microsystems) and an incubation chamber. The images were captured by a 20-MHz Cool SNAPHQ2 charge-coupled device camera (Roper Technologies), with a z-optical spacing of 0.2 μm. Metamorph software (Molecular Devices) was used to acquire z-series. Using this imaging device, one pixel corresponds to 64.5 nm.

Living cells in early exponential growth phase were observed in the culture medium, which was strictly maintained at 30 °C during slide mounting and observation. For each strain, large numbers of images of cells were acquired, for at least two different clones, in at least three independent experiments. Raw images were deconvolved.

In experiments to localize Rvs167-GFP and Rvs161-GFP (Supplementary Fig. S2), bud width and bud neck width were measured and the presence or absence of Rvs167-GFP or Rvs161-GFP signal at the tip of the small bud was scored for each cell.

In BiFC experiments, deconvolved stacks were overlayed without any modification of signals, then colocalizations of YFP and mRFP signals were scored for each cell plane-to-plane. A colocalization event corresponds to a group of pixels which present a signal visible to the naked eyes in both red and green channels. Manders M2 coefficients (defined as the ratio of the summed intensities of pixels from the red image for which the intensity in the green channel is above zero to the total intensity in the green channel) were calculated on deconvolved single planes, using the JacoP application of the Image J software^45^.

### Bgl2p-HA secretion assay

Volumes of cultures corresponding to 3 × 10^7^ cells were centrifuged to recover the cells, which were then resuspended in 0.1 M Tris–HCl pH 9.4, 10 mM DTT, and incubated with shaking for 10 min. The yeast cells were collected by centrifugation and spheroplasts were generated by incubation in 200 μL STC buffer (1 M sorbitol, 10 mM Tris–HCl pH 7.5 and 10 mM CaCl_2_) supplemented with 0.25 mg/mL Zymolyase 20 T (Valeant). Spheroplasts were collected by centrifugation at 100 × g for 10 min at 4 °C. 190 μL of the upper supernatant, containing the periplasmic Bgl2p-HA, and the pellet corresponding to internal Bgl2p-HA were diluted in appropriate volumes of Laemmli sample buffer and boiled. Volumes of samples corresponding to 1.8 × 10^6^ cells were separated by SDS–10% PAGE and blotted onto membranes, which were then probed with anti-HA antibodies (Roche). The upper part of the immunoblots was probed with anti-Cdc11p antibodies (Santa Cruz Biotechnology), both as a loading control for internal Bgl2p-HA samples and to check that there was no contamination with internal Bgl2p-HA for secreted Bgl2p-HA samples. The Bgl2p-HA signals were quantified by Image J Software. The values reported are means of three or four experiments with independent clones.

### Calcofluor staining

Exponentially growing cells were fixed in formaldehyde 4% (v/v) for two hours at 30 °C, harvested by centrifugation and washed in water, then resuspended in a calcofluor 1 mg/ml solution and incubated at room temperature for 5 minutes. After five washes in water, the cells were deposited between slide and coverslip for observation.

### Methodology and statistics

Immunoprecipitation experiments, Bgl2-HA secretion experiments were repeated three times in independent experiments. BiFC experiments were repeated at least two times in independent experiments. For each strain, 37 to 190 cells from both experiments were scored. The scoring was not blinded. Statistical analyses used were Chi^2^ test for the localization of Rvs161-GFP, Rvs167-GFP or Gyl1 at the tip of small buds (Figs. 1c and S1), for the localization of N-BAR proteins pairs at the tip of small buds (Fig. 4a,b) and for determination of budding polarity (Supplementary Fig. S5); Mann-Whitney test for the Bgl2-HA secretion experiments (Fig. 4c); Mann-Whitney-Wilcoxon rank sum test for the BiFC colocalization experiments (Figs. 5, 7 and S3).

## Supplementary information

Supplementary information

## Data Availability

All materials, methods and/or data sets are fully available upon request.
