# Supplementary material for: Three members of the yeast N-BAR proteins family form heterogeneous lattices in vivo and interact differentially with two RabGAP proteins
Source: Sci Rep. 2020 Feb 3;10:1698. doi: 10.1038/s41598-020-58606-2 (PMC6997364; doi:10.1038/s41598-020-58606-2)

Three members of the yeast N-BAR proteins family form heterogeneous lattices *in vivo* and interact differentially with two RabGAP proteins

Magali Prigent, Julien Chaillot, Hélène Tisserand, Emmanuelle Boy-Marcotte and Marie-Hélène Cuif

Supplementary Information

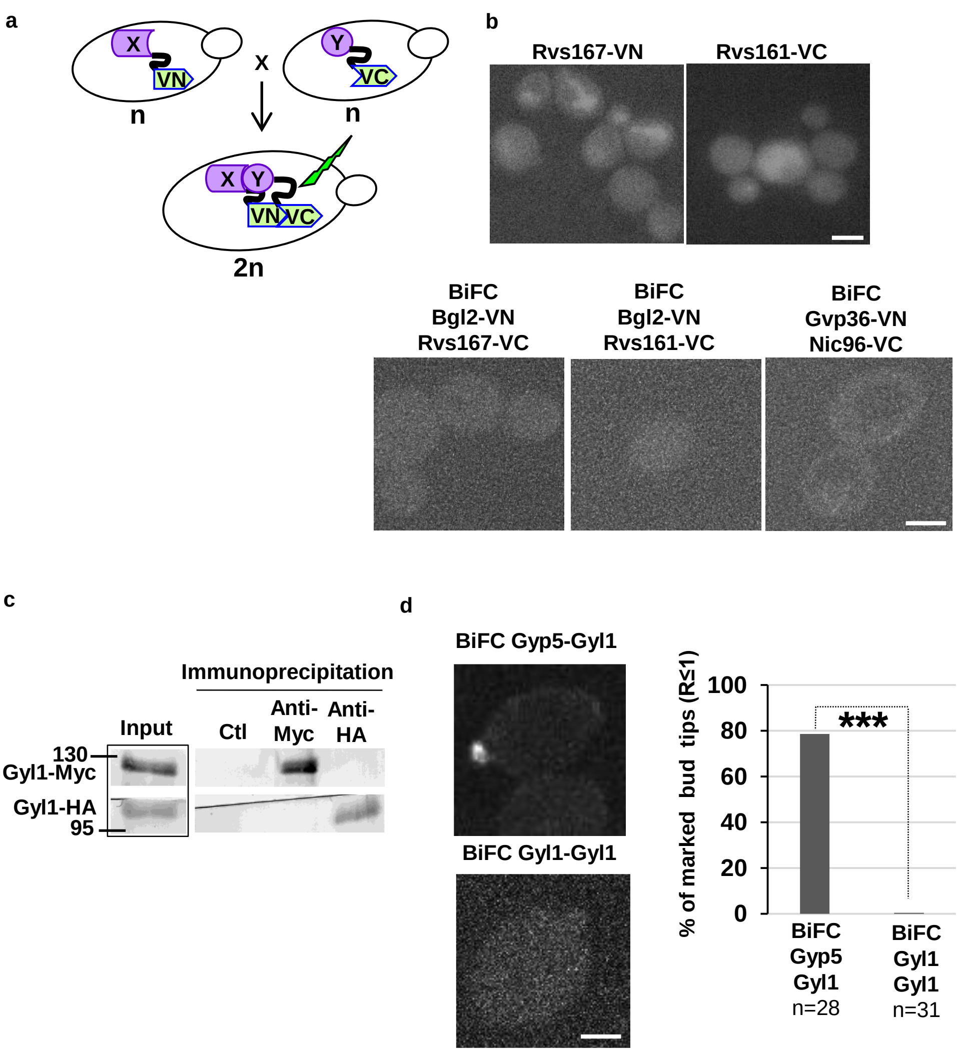

Supplementary figure S1. (a) The principle of the BiFC assay, as described by Sung et al. X and Y are proteins of interest; VN and VC indicate the N-terminal part and the C-terminal part of the Venus YFP, respectively. The green arrow indicates the emission of the BiFC signal. (b) Representative images of the BiFC signals observed in cells expressing only the Rvs167-VN or the Rvs161-VC fusion proteins or co-expressing the Bgl2-VN/Rvs167-VC, Bgl2-VN/Rvs161-VC, Gvp36-VN/Nic96-VC fusion proteins, as indicated. The scale bar, 3µm, is applicable to all the cells of the row.(c) Immunoprecipitation experiments were performed on total extracts of log-phase diploid cells co-expressing Gyl1-Myc and Gyl1-HA. Membranes were cut at the appropriate sizes for incubation with anti-Myc and anti-HA antibodies. Parts of the film were grouped. The full length film is available in Supplementary Figure S6. The image shown is representative of three independent experiments. (d) Representative images of the BiFC signal detected in small-budded cells co-expressing Gyp5-VN and Gyl1-VC (up) or Gyl1-VN and Gyl1-VC (down). Scale bar, 3µm. Small bud tips were scored for BiFC signals corresponding to each indicated pair of N-BAR proteins. n is the number of small bud tips scored. *** indicates a significant difference between observed distributions (p<0.001,Chi[2] test).

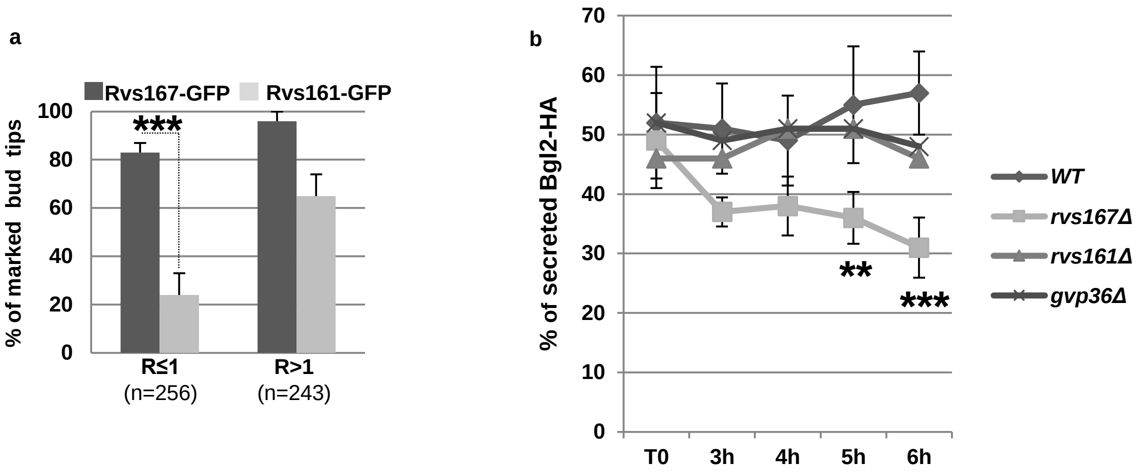

Supplementary figure S2. (a) Rvs161 is frequently absent from the tip of small buds : percentages of small-budded cells displaying a patch of Rvs167p-GFP or Rvs161-GFP at the bud tip, according to the bud width to bud-neck width ratio R. The values reported are the mean ± s.e.m. of two independent experiments. *** indicates a significant difference between observed distributions (p<0.001,Chi^2 test) (b) Bgl2-HA secretion is unaffected in *rvs161Δ* and *gvp36Δ* cultures enriched in small-budded cells. WT, *rvs167Δ*, *rvs161Δ* and *gvp36Δ* expressing Bgl2-HA under the control of the GAL promoter were cultured at 13°C in the presence of 0.1% glucose, then transferred to 2% galactose. The amounts of secreted and internal Bgl2-HA were assessed by western blotting. The percentage of secreted Bgl2-HA is shown. The values reported are the mean ± s.e.m. of three or four experiments on independent clones. The stars indicate a significant difference between observed values for mutant strains and the WT strain at each time-point (** p<0.01, *** p<0.001, Mann-Whitney test).

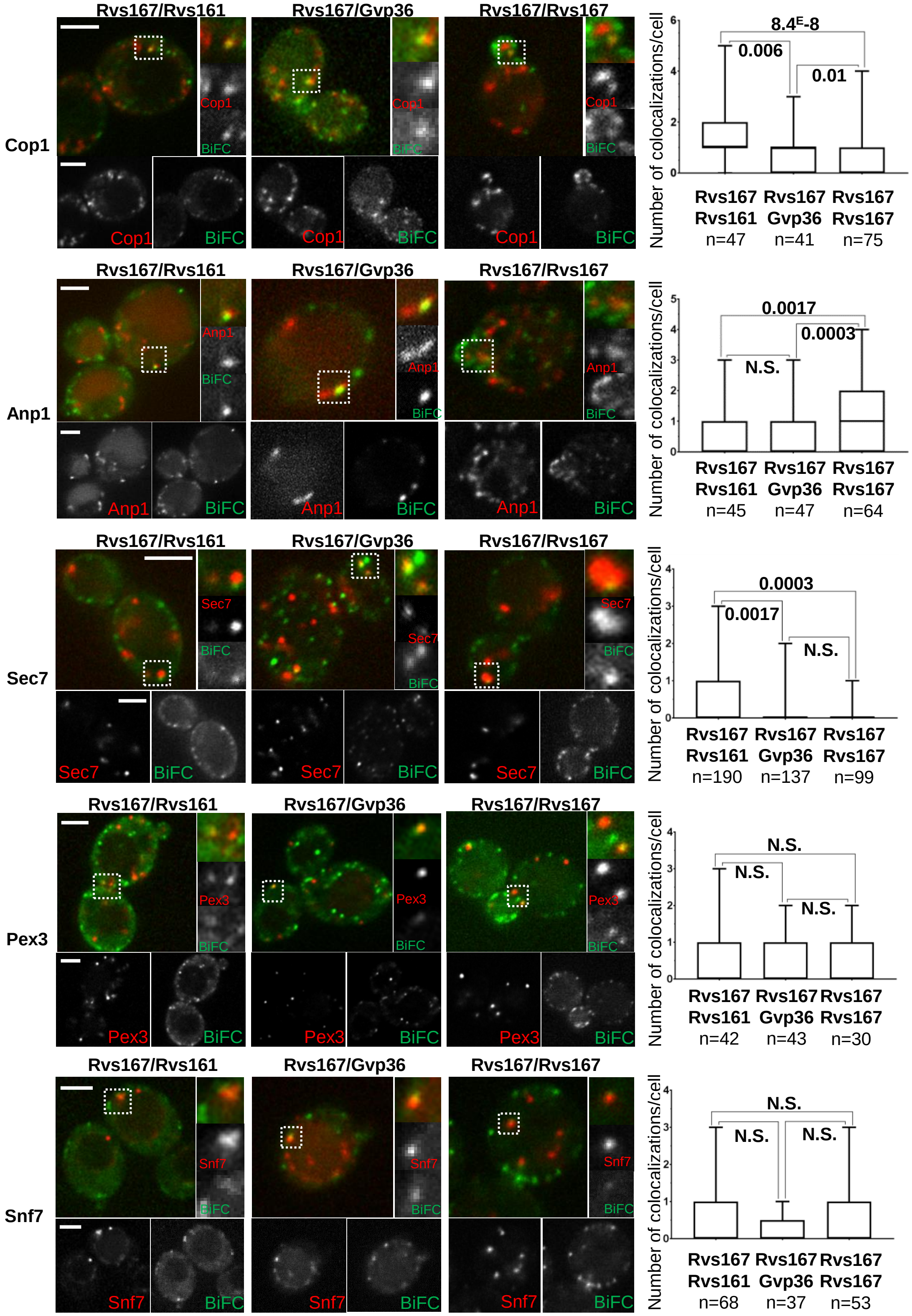

Supplementary Figure S3. Colocalization of the BiFC signals generated by pairs of N-BAR proteins with mRFP markers. Representative images of colocalization events in cells co-expressing each pair of N-BAR proteins and the indicated mRFP marker. One single plane from a z-stack is shown. The scale bar, 3µm, is applicable to all the cells of the panel. For each panel, quantifications and statistical analysis of the results are shown. Detailed quantification of the colocalization events are shown in Supplementary Table S2 online. The box plots indicate the mean ±S.D., minimal and maximal values. n is the number of cells scored. Statistical differences between the observed distributions were tested by the Wilcoxon rank sum test. The indicated p values represent the probability of identity of the two distributions. N.S. indicates a p value > 0.05.

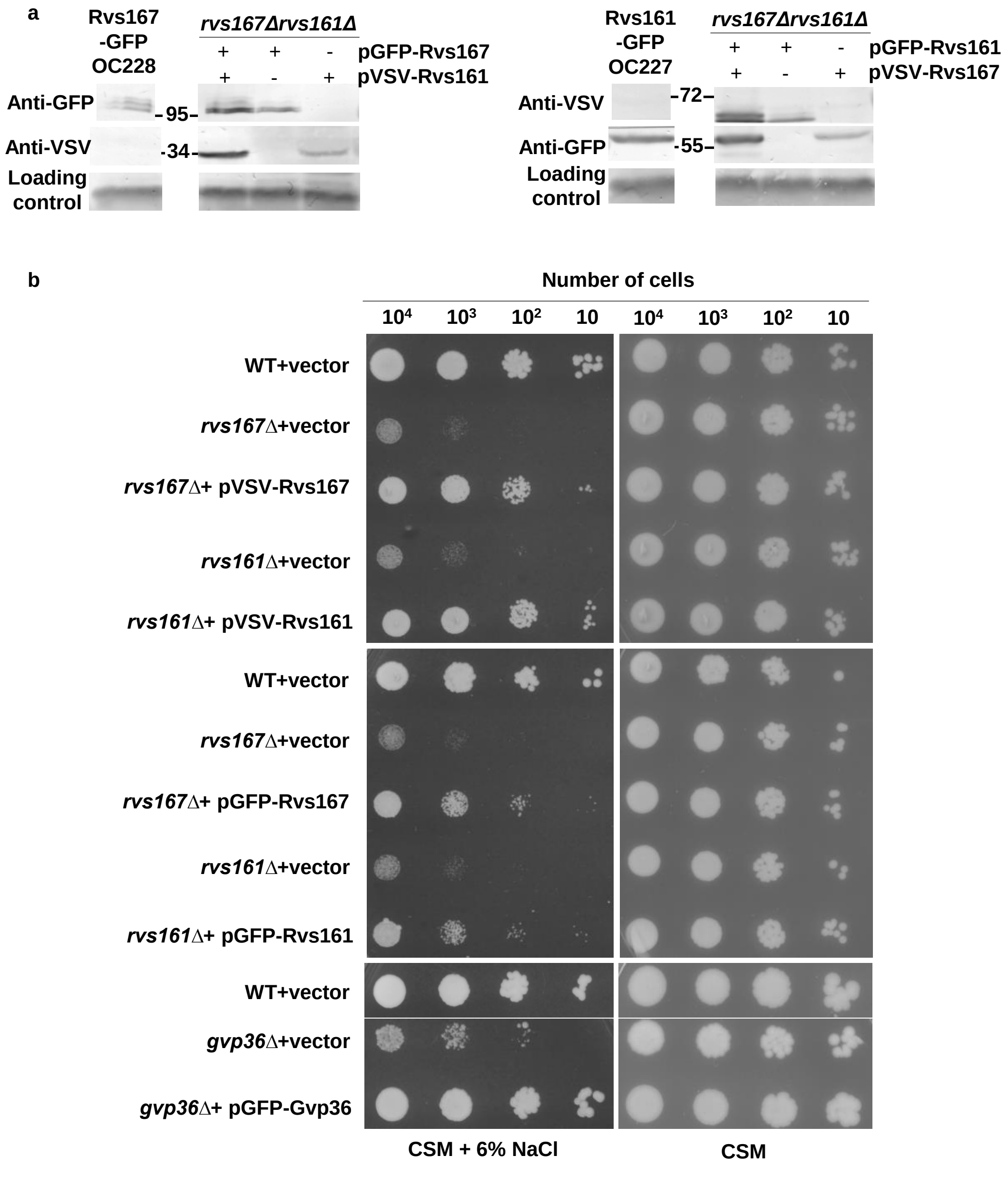

Supplementary figure S4. Expression, stability and functionality of the N-BAR fusion proteins used in this study. (a) Protein extracts from *rvs167Δ161Δ* cells transformed with plasmids encoding the pGFP-Rvs167, p-VSV RVS167, pGFP-RVS161 or pVSV-Rvs161 fusion proteins were resolved by SDS-PAGE and probed with anti-GFP or anti-VSV antibody. The controls shown correspond to $1x10^7$ locus-tagged Rvs167-GFP or Rvs161-GFP cells. Parts of the film were grouped. The full length film is available in Supplementary Figure S6(b) Cells transformed with the indicated plasmids were grown to log phase, diluted, dropped on CSM or CSM+6%NacCl as indicated and cultured for 2 days at 30°C.

a

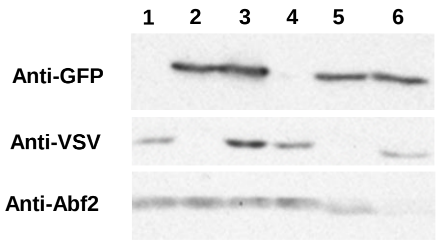

1- n WT+ pVSV-RVS161
2- n WT + pGFP-GVP36
3- n WT + pVSV-RVS161 + pGFP-GVP36
4- 2n WT+ pVSV-RVS161
5- 2n WT+ pGFP-GVP36
6- 2n WT+ pVSV-RVS161 + pGFP-GVP36

b

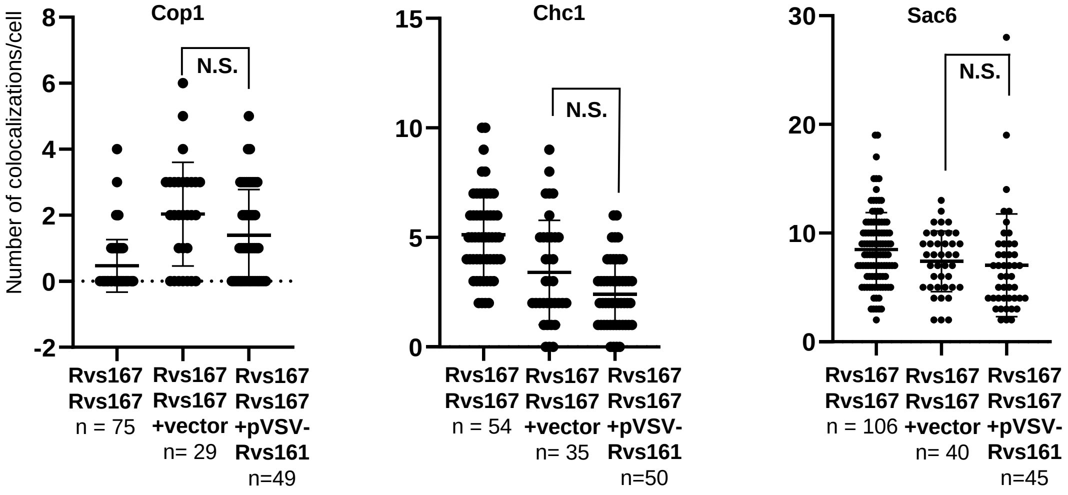

c

	n	% bipolar	% axial	% random
2n WT+vector	57	77,2	19,3	3,5
2n WT+ pVSV-RVS161	58	72,4	22,4	5,2
2n WT+vector	69	75,4	20,3	4,3
2n WT+pGFP-GVP36	66	69,7	27,3	3,0
2n WT+vectors	73	74,0	24,7	1,4
2n WT+pVSV-RVS161+pGFP-GVP36	63	68,3	27,0	4,8

d

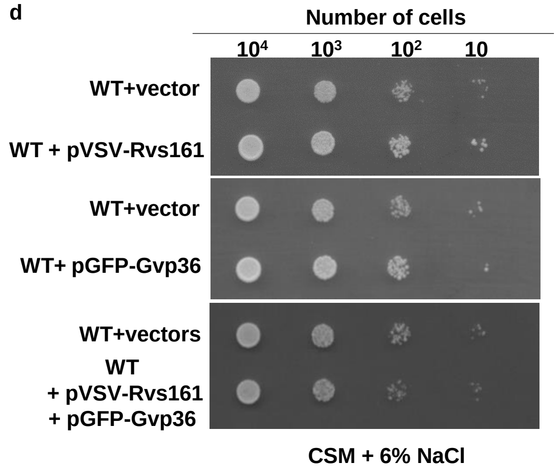

Supplementary Figure S5. (a) Cells were transformed by the indicated combinations of plasmids and the expression of GFP-Gvp36 and VSV-Rvs161 was probed by immunoblot. Membranes were cut at the appropriate sizes for incubation with anti-VSV and anti-GFP antibodies. Parts of the film were grouped. The full length film is available in Supplementary Figure S6. (b) Quantifications and statistical analysis of the colocalization of the BiFC signals generated by the Rvs167/Rvs167 pair with mRFP markers in cells overexpressing VSV-Rvs161, in three independent experiments.The box plots indicate the mean ±S.D., minimal and maximal values. n is the number of cells scored. Statistical differences between the observed distributions were tested by the Wilcoxon rank sum test. The indicated p values represent the probability of identity of the two distributions. N.S. indicates a p value > 0.05. (c) The budding polarity of diploid WT cells overexpressing the indicated combination ofN-BAR proteins was scored after calcofluor staining. No significant difference was found between the different categories (Chi^2 test). (d) Strains harboring the indicated combinations of plasmids were grown to log phase, diluted, dropped on CSM+6%NacCl and cultured for 2 days at 30°C. Representative image of two independent experiments.

Strain	Genotype	Source
**OC 1**	*MATa ura3Δ0 his3Δ1 leu2Δ0 met15Δ0 lys2Δ0*	Chesneau, 2004
**OC 2**	*MAT α ura3Δ0 his3Δ1 leu2Δ0*	Chesneau, 2004
**OC 213**	*MATa/α GYL1-MYC-HIS3MX6 GYL1-HA-HIS3MX6 ura3Δ0 his3Δ1 leu2Δ0 met15Δ0*	
**OC 226**	*MATα rvs161:: KanMX4 ura3Δ0 his3Δ1 leu2Δ0 lys2Δ0*	Winzeler, 1999
**OC 227**	*MATa RVS161-GFP(S65T)-HIS3MX6 ura3Δ0 his3Δ1 leuΔ0 met15Δ0*	Huh, 2003
**OC 228**	*MATa RVS167-GFP(S65T)-HIS3MX6 ura3Δ0 his3Δ1 leuΔ0 met15Δ0*	Huh, 2003
**OC 246**	*MATa gvp36::KanMX4 his3Δ1 leu2Δ0 met15Δ0 ura3Δ0*	Winzeler, 1999
**OC222b**	*MATa rvs167::KanMX4 ura3Δ0 his3Δ1 leuΔ0 met15Δ0*	Winzeler, 1999
**OC271**	*MATa RVS167-VN-HIS3MX6 ura3Δ0 his3Δ1 leu2Δ0 met15Δ0 lys2Δ0*	This study
**OC273**	*MATa RVS161-VN-HIS3MX6 ura3Δ0 his3Δ1 leu2Δ0 met15Δ0 lys2Δ0*	This study
**OC277**	*MATa GYP5-VN::HIS3MX6 ura3Δ0 his3Δ1 leu2Δ0 met15Δ0 lys2Δ0*	This study
**OC279**	*MATα RVS167-VC-KanMX6 ura3Δ0 his3Δ1 leu2Δ0*	This study
**OC281**	*MATα RVS161-VC-KanMX6 ura3Δ0 his3Δ1 leu2Δ0*	This study
**OC284**	*MATα GYP5-VC::KanMX6 ura3Δ0 his3Δ1 leu2Δ0*	This study
**OC287**	*MATα GVP36-VC-KanMX6 ura3Δ0 his3Δ1 leu2Δ0*	This study
**OC308**	*MATα GYL1-3xHA-HIS3MX6 GYP5-13xMYC-HIS3MX6 rvs161::KanMX4, rvs167::KanMX4 ura3Δ0 his3Δ1 leu2Δ0 met15Δ0*	This study
**OC320**	*MATa GYL1-3xHA-HIS3MX6 GYP5-13xMYC-HIS3MX6 gvp36::KanMX4 rvs167::KanMX4 ura3Δ0 his3Δ1 leu2Δ0*	This study
**OC353**	*MATa GVP36-VN-HIS3MX6 ura3Δ0 his3Δ1 leu2Δ0 met15Δ0 lys2Δ0*	This study
**OC382**	*MATα BGL2-VC-KanMX6 ura3Δ0 his3Δ1 leu2Δ0*	This study
**OC383**	*MATa GYL1-HA-HIS3MX6 GYP5-MYC-HIS3MX6 rvs161::KanMX4 rvs167::KanMX4 gvp36::KanMX4 ura3Δ0 his3Δ1 leu2Δ0*	This study
**OC385**	*MATa ANP1-mRFP-KanMX6 RVS167-YFP-VN-HIS3MX6 his3Δ1 ura3Δ0 leu2Δ0 lys2Δ0 met15Δ0?*	This study
**OC386**	*MATa CHC1-mRFP-KanMX6 RVS167-YFP-VN-HIS3MX6 his3Δ1 ura3Δ0 leu2Δ0 lys2Δ0 met15Δ0?*	This study
**OC387**	*MATa COP1-mRFP-KanMX6 RVS167-YFP-VN-HIS3MX6 his3Δ1 ura3Δ0 leu2Δ0 lys2Δ0 met15Δ0?*	This study
**OC389**	*MATa PEX3-mRFP-KanMX6 RVS167-YFP-VN-HIS3MX6 his3Δ1 ura3Δ0 leu2Δ0 lys2Δ0 met15Δ0?*	This study
**OC390**	*MATa SAC6-mRFP-KanMX6 RVS167-YFP-VN-HIS3MX6 his3Δ1 ura3Δ0 leu2Δ0 lys2Δ0 met15Δ0?*	This study
**OC391**	*MATa SEC13-mRFP-KanMX6 RVS167-YFP-VN-HIS3MX6 his3Δ1 ura3Δ0 leu2Δ0 lys2Δ0 met15Δ0?*	This study
**OC392**	*MATa SNF7-mRFP-KanMX6 RVS167-YFP-VN-HIS3MX6 his3Δ1 ura3Δ0 leu2Δ0 lys2Δ0 met15Δ0?*	This study
**OC402**	*MATa BGL2-VN-HIS3MX6 ura3Δ0 his3Δ1 leu2Δ0 met15Δ0 lys2Δ0*	This study
**OC404**	*MATα NIC96-VC-KanMX6 ura3Δ0 his3Δ1 leu2Δ0*	This study
**OC412**	*MATa CHC1-mRFP-KanMX6 RVS161-YFP-VN-HIS3MX6 his3Δ1 ura3Δ0 leu2Δ0 lys2Δ0 met15Δ0?*	This study
**OC413**	*MATa PEX3-mRFP-KanMX6 RVS161-YFP-VN-HIS3MX6 his3Δ1 ura3Δ0 leu2Δ0 lys2Δ0 met15Δ0?*	This study
**OC416**	*MATa ANP1-mRFP-KanMX6 RVS161-YFP-VN-HIS3MX6 his3Δ1 ura3Δ0 leu2Δ0 lys2Δ0 met15Δ0?*	This study
**OC421**	*MATa SEC13-mRFP-KanMX6 Gvp36-YFP-VN-HIS3MX6 his3Δ1 ura3Δ0 leu2Δ0 lys2Δ0 met15Δ0?*	This study
**OC423**	*MATa COP1-mRFP-KanMX6 Gvp36-YFP-VN-HIS3MX6 his3Δ1 ura3Δ0 leu2Δ0 lys2Δ0 met15Δ0?*	This study
**OC424**	*MATa SAC6-mRFP-KanMX6 Gvp36-YFP-VN-HIS3MX6 his3Δ1 ura3Δ0 leu2Δ0 lys2Δ0 met15Δ0?*	This study
**OC425**	*MATa PEX3-mRFP-KanMX6 Gvp36-YFP-VN-HIS3MX6 his3Δ1 ura3Δ0 leu2Δ0 lys2Δ0 met15Δ0?*	This study
**OC426**	*MATa CHC1-mRFP-KanMX6 Gvp36-YFP-VN-HIS3MX6 his3Δ1 ura3Δ0 leu2Δ0 lys2Δ0 met15Δ0?*	This study
**OC430**	*MATa RVS167-VN::HIS3MX6 SEC7-mCHERRY-KanR ura3Δ0 his3Δ1 leu2Δ0 met15Δ0 lys2Δ0*	This study
**OC434**	*MATa RVS167-VN::HIS3MX6 RTN1-mCHERRY-KanR ura3Δ0 his3Δ1 leu2Δ0 met15Δ0 lys2Δ0*	This study
**OC460**	*MATa COP1-mRFP-KanMX6 RVS167-YFP-VN-HIS3MX6 rvs161::hph his3Δ1 ura3Δ0 leu2Δ0 lys2Δ0 met?*	This study
**OC462**	*MATa CHC1-mRFP-KanMX6 RVS167-YFP-VN-HIS3MX6 rvs161::hph his3Δ1 ura3Δ0 leu2Δ0 lys2Δ0 met?*	This study
**OC463**	*MATa Sac6-mRFP-KanMX6 RVS167-YFP-VN-HIS3MX6 rvs161::hph his3Δ1 ura3Δ0 leu2Δ0 lys2Δ0 met?*	This study

Supplementary table S1. Strains used in this study

		BiFC pairs		
		Rvs167/Rvs161	Rvs167/Gvp36	Rvs167/Rvs167
**Sac6**	% of cells with colocalization	100% (n=60)	55% (n=133)	100% (n=102)
	number of colocalizations/cell	1 to 8, m=4	1 to 4, m=1.5	3 to 19, m=8.4
	p value Wilcoxon rank sum test		1,70E-22	1,10E-17
	Arbitrary units (lane1xlane2)	400	82	840
	% of the total in arbitrary units	**30,3**	**6,2**	**63,5**
**Chc1**	% of cells with colocalization	57% (n=42)	36% (n=74)	100% (n=53)
	number of colocalizations/cell	1 to 3, m=2	1 to 3, m=1.3	1 to 10, m=5
	p value Wilcoxon rank sum test		0.004	4,20E-15
	Arbitrary units (lane1xlane2)	114	46	500
	% of the total in arbitrary units	**17,3**	**7,0**	**75,8**
**Sec13**	% of cells with colocalization	100% (n=53)	34% (n=66)	100% (n=51)
	number of colocalizations/cell	1 to 7, m=3.4	1 to 3, m=1.3	1 to 8, m= 4
	p value Wilcoxon rank sum test		9,60E-14	0.088 NS
	Arbitrary units (lane1xlane2)	340	44	400
	% of the total in arbitrary units	**43,4**	**5,6**	**51,0**
**Rtn1**	% of cells with colocalization	91% (n=96)	32% (n=104)	51% (n=152)
	number of colocalizations/cell	1 to 6, m=2.4	1 to 3, m=1.4	1 to 3, m=1.4
	p value Wilcoxon rank sum test		9,90E-21	3,40E-18
	Arbitrary units (lane1xlane2)	218	44	71
	% of the total in arbitrary units	**65,5**	**13,2**	**21,3**
**Cop1**	% of cells with colocalization	80% (n=47)	58% (n=41)	33% (n=75)
	number of colocalizations/cell	1 to 5, m=1.4	1 to 3, m=0,8	1 to 4, m=0,4
	p value Wilcoxon rank sum test		0,006	8,40E-08
	Arbitrary units (lane1xlane2)	144	48	15
	% of the total in arbitrary units	**69,6**	**23,2**	**7,2**
**Anp1**	% of cells with colocalization	35% (n=45)	25% (n=47)	57% (n=64)
	number of colocalizations/cell	1 to 3, m=1.4	1 to 3, m=1.2	1 to 4, m=1.7
	p value Wilcoxon rank sum test		0.249 NS	0.017
	Arbitrary units (lane1xlane2)	49	30	85
	% of the total in arbitrary units	**29,9**	**18,3**	**51,8**
**Sec7**	% of cells with colocalization	28% (n=190)	13% (n=137)	10% (n=99)
	number of colocalizations/cell	1 to 3, m=1.4	1 to 2, m=1.2	1
	p value Wilcoxon rank sum test		0.01	0.002
	Arbitrary units (lane1xlane2)	39	15	10
	% of the total in arbitrary units	**60,9**	**23,4**	**15,6**
**Pex3**	% of cells with colocalization	28% (n=42)	30% (n=43)	40% (n=30)
	number of colocalizations/cell	1 to 3, m=1.2	1 to 2, m=1.3	1 to 2, m=1
	p value Wilcoxon rank sum test			
	Arbitrary units (lane1xlane2)	33	39	40
	% of the total in arbitrary units	**29,5**	**34,8**	**35,7**
**Snf7**	% of cells with colocalization	35% (n=68)	24% (n=37)	32% (n=53)
	number of colocalizations/cell	1 to 3, m=1.1	1	1 to 3, m=1,3
	p value Wilcoxon rank sum test			
	Arbitrary units (lane1xlane2)	33	39	40
	% of the total in arbitrary units	**32,0**	**37,9**	**38,8**

Supplementary table S2. Quantification of the colocalization of the BiFC signals generated by pairs of N-BAR proteins with mRFP markers. mRFP and YFP signals were acquired in exponentially growing cells of each strain, and the number of colocalization events was scored plane-to-plane in each cell. For each marker, the first line indicates the percentage of cells in which at least one colocalization event was observed and, in brackets, the number of cells scored. The second line indicates the minimal, maximal and mean number of colocalization events per cell. The third line reports the result of multiplication of the values of the first and second lines, serving as a value in arbitrary units reflecting the abundance of interactions of each pair of N-BAR proteins colocalized with each mRFP marker; these values are used for the graphical representation in Fig. 6a.

Supplementary figure S6

Fig.1b

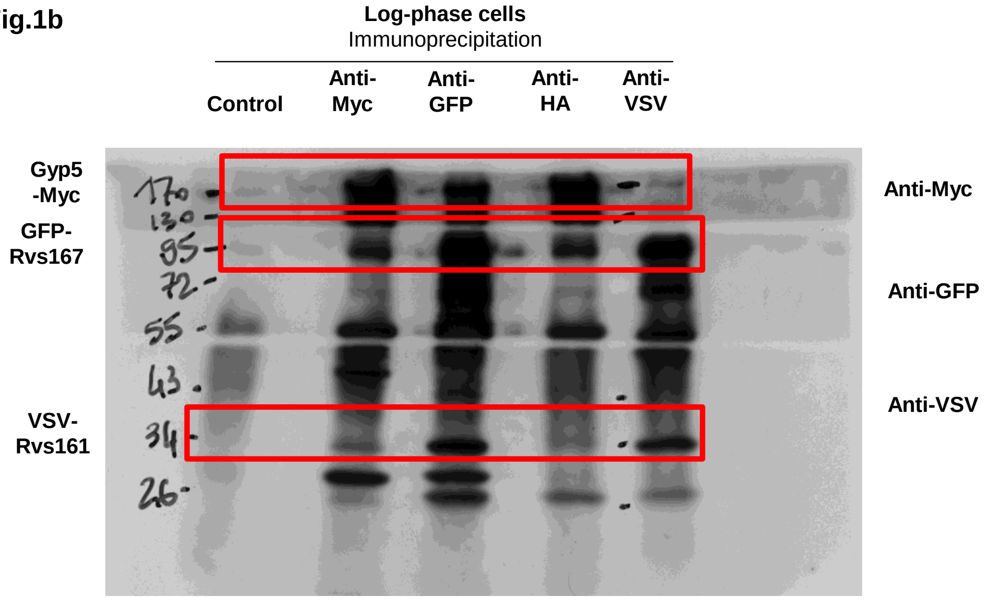

Fig.1c

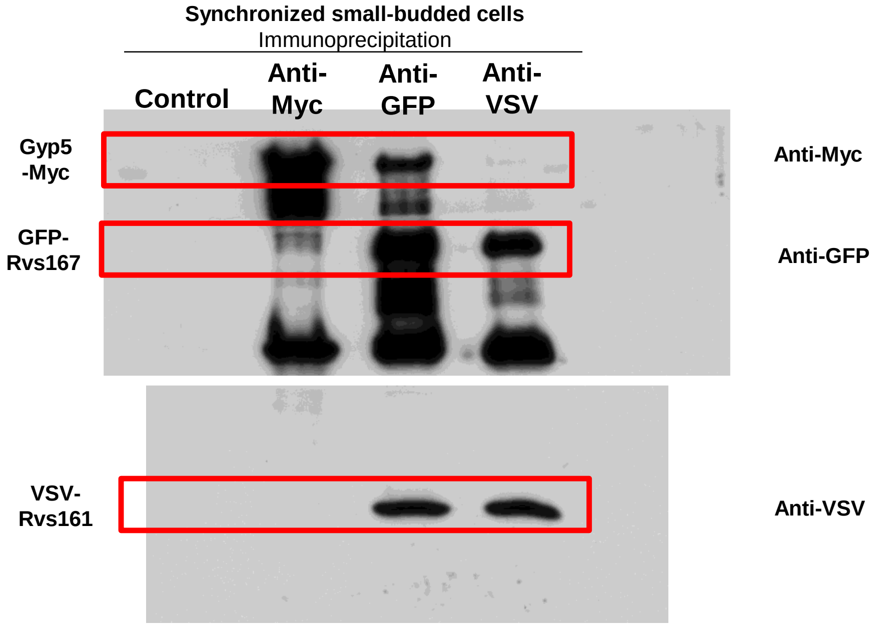

Supplementary figure S6 continued

Fig. 2a

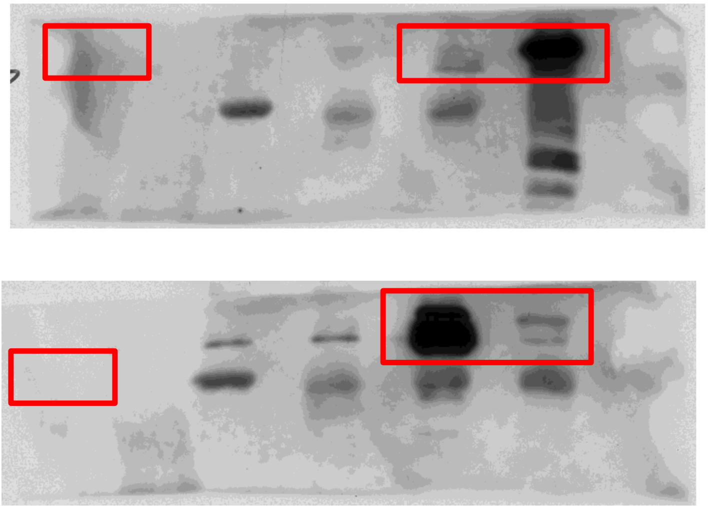

Fig. 2b

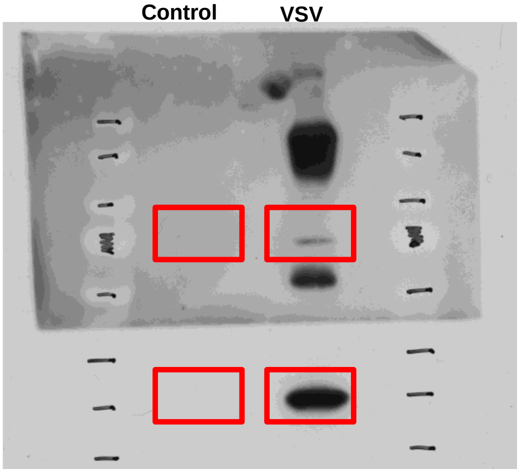

Supplementary figure S6 continued

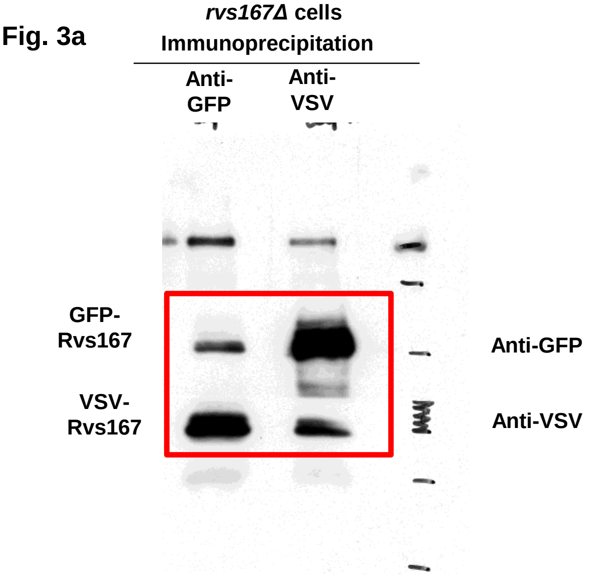

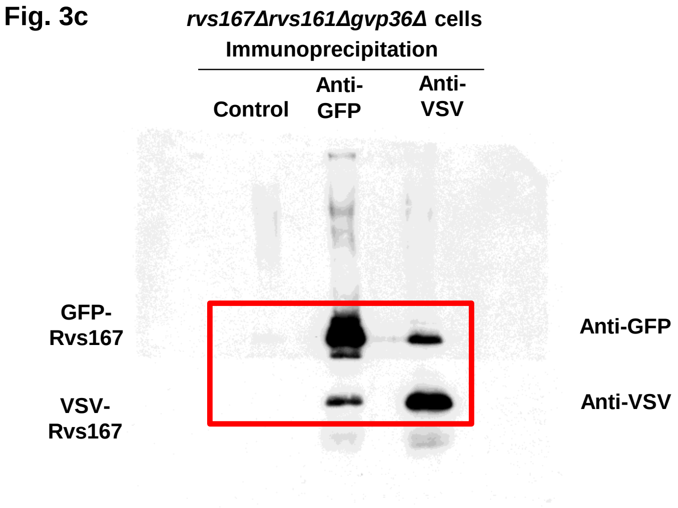

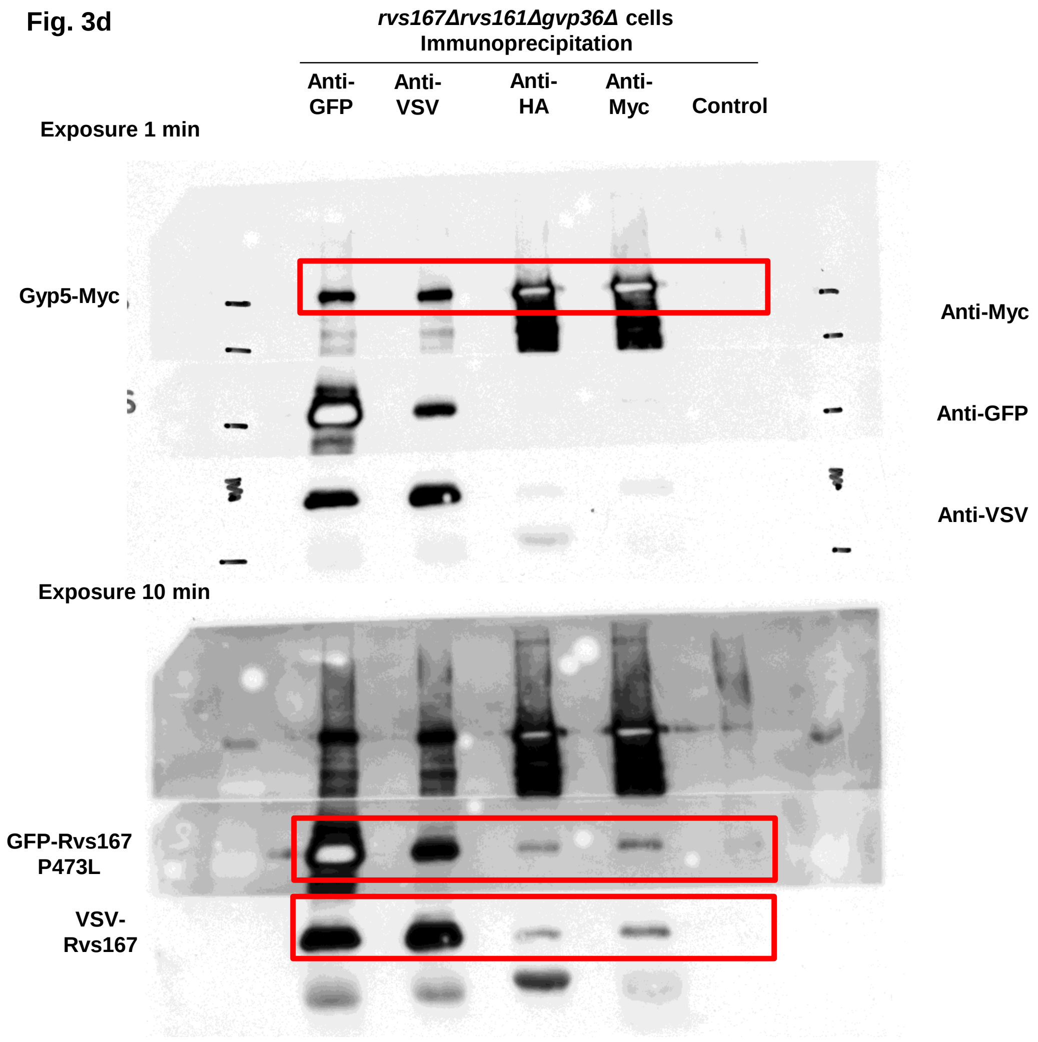

Supplementary figure S6 continued

Fig. S1d

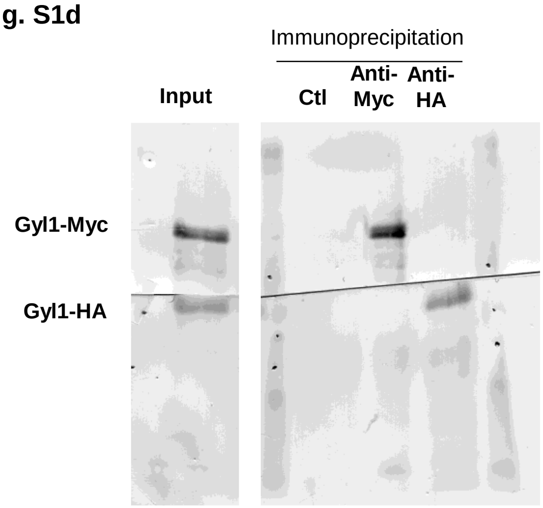

Fig. S5a

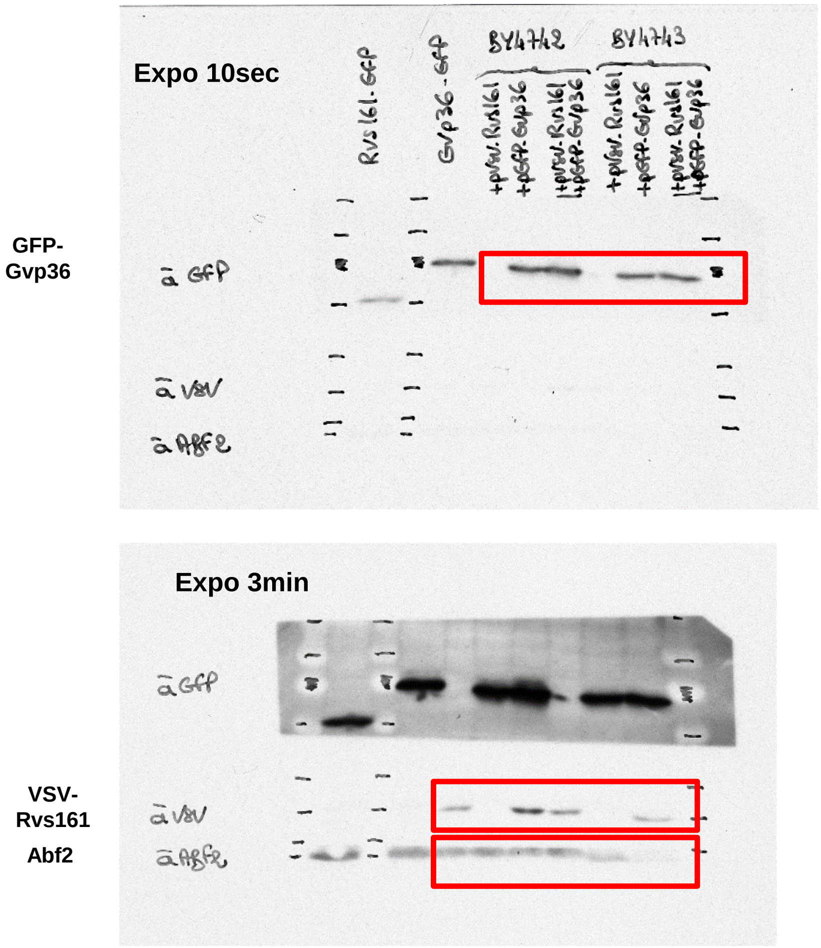

Supplement: Supplementary file 1 — Supplementary information [file 41598_2020_58606_MOESM1_ESM.pdf]
